# Differential expression of microRNAs in decidua-derived mesenchymal stem cells from patients with pre-eclampsia

**DOI:** 10.1186/s12929-014-0081-3

**Published:** 2014-08-19

**Authors:** Guangfeng Zhao, Xue Zhou, Shiwen Chen, Huishuang Miao, Hongye Fan, Zhiqun Wang, Yali Hu, Yayi Hou

**Affiliations:** 1 Department of Obstetrics and Gynecology, Nanjing Drum Tower Hospital, Nanjing University Medical School, Nanjing 210008, China; 2 Immunology and Reproductive Biology Laboratory, Medical School & State Key Laboratory of Pharmaceutical Biotechnology, Nanjing University, Nanjing 210093, China

**Keywords:** Pre- eclampsia, Mesenchymal stem cells, miRNAs, Microarray

## Abstract

**Background:**

Mesenchymal stem cells (MSCs) at maternal-fetal interface are considered to play an important role in the pathogenesis of pre-eclampsia (PE). microRNAs (miRNAs) also have an important influence on differentiation, maturation, and functions of MSCs. Our aim in this study was to determine the differential expression of miRNAs in decidua-derived MSCs (dMSCs) from severe PE and normal pregnancies.

**Results:**

miRNA expression profiles in dMSCs from five patients with severe PE and five healthy pregnant women were screened using microarray. Then, bioinformatic analysis of the microarray results was performed. Out of 179 differentially expressed miRNAs, 49 miRNAs had significant (p < 0.05) differential expression of ≥ 2.0-fold changes, including 21 up-regulated and 28 down-regulated. miRNA-Gene-network and miRNA-Gene ontology (GO) -network analyses were performed. Overall, 21 up-regulated and 15 down-regulated miRNAs showed high degrees in these analyses. Moreover, the significantly enriched signaling pathways and GOs were identified. The analyses revealed that pathways associated with cell proliferation, angiogenesis, and immune functions were highly regulated by the differentially expressed miRNAs, including Wnt signaling pathway, mitogen-activated protein kinase signaling pathway, transforming growth factor beta signaling pathway, T-cell receptor signaling pathway, and B cell receptor signaling pathway. Four miRNA predicted target genes, vascular endothelial growth factor A (VEGFA), indoleamine 2,3-dioxygenase, suppression of cytokine signaling 3, and serine/threonine protein phosphatase 2A 55 kDa regulatory subunit B α isoform (PPP2R2A) were all decreased in dMSCs from patients with PE. Furthermore, the physiological roles of miR-16 and miR-136 in the down-regulation of VEGFA and PPP2R2A, respectively, were confirmed through reporter assays.

**Conclusions:**

These findings suggest that miRNAs in dMSCs may be important regulatory molecules in the development of PE.

## Background

Pre-eclampsia (PE) affects approximately 5% of pregnancies and remains a leading cause of maternal and neonatal mortality and morbidity in the world [1]. While much research has been devoted toward this topic, the cause of PE still remains elusive [2]. Previous studies indicate that the imbalanced immune system in the maternal–fetal interface may be one cause of PE [1],[3],[4]. Maternal immune maladaptation toward the feto-placental district is a cause for the development of defective trophoblast and related maternal-placental pathological anomalies, such as PE [5],[6]. Moreover, studies have shown that PE results in a shift in angiogenesis and anti-angiogenic factors toward a maladaptive placental circulation [7],[8]. It indicates that abnormality in placental vascular remodeling is also a likely pathogenesis of PE [9],[10].

Mesenchymal stem cells (MSCs) are multi-potent progenitor cells, which can differentiate into various cell types, such as osteoblasts, adipocytes and chondroblasts, and are easily expanded and stored ex vivo [11],[12]. MSCs are the focus of intensive efforts worldwide directed not only at elucidating their nature and unique properties but also in developing cell-based therapies for a diverse range of diseases [13]. They are considered to be immune-privileged and shown to exert a strong inhibitory effect on other immune cells [14]–[17]. Over 300 clinical trials related to tissue repair and immune conditions have been conducted in treatment with MSCs because of its immunosuppressive properties [18]–[20]. Moreover, MSCs are promising tools for treating diseases such as myocardial infarction and stroke due to their ability to promote endogenous angiogenesis and neurogenesis through a variety of secreted factors [21].

The maternal-fetal interface is an important source of MSCs [22]–[24]. Aberrant levels of cytokines were observed in placenta-derived MSCs from patients with PE, and higher levels of MSC negative markers were found in the placentas from patients with PE [25],[26]. These findings suggest that MSCs may contribute to pathogenesis of PE. Therefore, investigation of the immune-modulatory, pro-angiogenic, and anti-inflammatory properties of decidua-derived MSCs (dMSCs) may open new perspectives into the understanding of PE [5].

MicroRNAs (miRNAs) are small noncoding RNAs that control gene expression by binding to target messenger RNAs (mRNAs) and thereby inducing translational repression or degradation of mRNAs [27]. miRNAs contribute to embryonic development and tissue homeostasis but even more profoundly regulate pathophysiological processes [28],[29]. miRNAs also have an important influence on differentiation, maturation, and functions of stem cells [30],[31]. Moreover, it was reported that differential expression of miRNAs has been observed in placentas from patients with PE [32],[33]. Therefore, it was hypothesized that miRNAs may be involved in the pathogenesis of PE by regulating dMSCs.

In this study, for a better understanding of the pathogenesis of the PE, the miRNA expression profiles of dMSCs from patients with PE and healthy pregnant women were analyzed using microarray. Bioinformatic analysis of the microarray results was performed. The results showed that the differences in miRNAs and their regulated signaling pathways exist in dMSCs from healthy pregnant women and patients with PE. The expression of target genes of differentially expressed miRNAs in dMSCs from patients with PE was also detected. These findings have important implications for revealing the pathogenesis of PE.

## Methods

### Patients and tissue samples

Human decidua tissues from patients with PE and age-matched normotensive controls were collected in the Department of Gynecology and Obstetrics of the Affiliated Drum Tower Hospital of Nanjing University Medical School (Nanjing, China). The hospital’s ethics committee approved the consent forms and the protocol for evaluating the tissue. Written consent form was received from each patient prior to surgery. PE was defined as the presence of hypertension and proteinuria beyond the 20th week of pregnancy. Elevation in blood pressure with systolic blood pressure > 140 mm Hg or diastolic pressure > 90 mm Hg was considered hypertensive. All deciduas were obtained at the time of cesarean section. Twenty pregnant women who had complications of severe late-onset PE with delivery occurring after 34 weeks and 20 women with normal term pregnancies as the control group were recruited. The relevant clinical characteristics of the patients are presented in Table 1. Any complications of pregnancy such as multiple pregnancies including twins, fetal structural or genetic anomalies, presence of maternal chronic hypertension, hemolysis, elevated liver enzyme levels, the HELLP syndrome, cardiovascular disease, renal disease, hepatic disease, diabetes, or other infectious disease were criteria for exclusion.

**Table 1:** Clinical characteristics of study population

	**PE (N = 20)**	**Control (N = 20)**	**p-Value**
Age, years	29.2 ± 1.4	28.9 ± 1.2	NS
Gestational age at delivery, weeks	37.3 ± 0.3	38.8 ± 0.5	NS
% of primiparae	11 (55%)	14 (70%)	NS
Body mass index, kg/m^2^	28.3 ± 0.9	27.9 ± 1.1	NS
Systolic blood pressure, mm Hg	160.4 ± 4.7	118.3 ± 3.4	<0.05
Diastolic Blood pressure, mm Hg	110.4 ± 4.1	82.5 ± 3.8	<0.05
Proteinuria, mg/24 h	2108 ± 30.4	0	<0.05
Alanine aminotransferase, U/L	35.3 ± 2.3	31.8 ± 3.1	NS
Blood urea nitrogen, mmol/L	3.9 ± 0.4	3.7 ± 0.6	NS
Platelet, ×10^9^/L	160.4 ± 32.1	194.3 ± 30.8	NS
Birth weight, g	2901 ± 183	3278 ± 203	NS
Placenta weight, g	476 ± 45	513 ± 52	NS

PE, pre-eclampsia; NS, non-significant.

### Isolation and culture of MSCs from deciduas

The decidua tissues were cut into 1–2 mm^3^ fragments and incubated in an enzyme cocktail (5 U/mL hyaluronidase, 125 U/mL collagenase and 50 U/mL dispase; Sigma, St Louis, MO) for 90–120 min with gentle agitation at 37°C. This tissue was then crushed with forceps to release individual cells, and large pieces of tissue were removed. The cells were pelleted by centrifugation at 250 *g* for 5 min, resuspended in fresh medium containing Dulbecco’s modified Eagle’s medium (DMEM) / F12 (Gibco, Grand Island, NY) and 20% fetal bovine serum and transferred to six well plates. Cells were incubated at 37°C in an incubator with 5% CO_2_ at saturating humidity. When cells reached 70–80% confluence or when numerous colonies were observed, the cells were detached using 0.25% trypsin/ethylenediaminetetraacetic acid (Invitrogen, Carlsbad, CA, USA), and the trypsin was inactivated using DMEM/F12. The culture medium was replaced every 3 or 4 days.

### Flow cytometry

After passages 2–4, the specific surface antigens of dMSCs in the cultures were detected by flow cytometry analysis. The following mouse anti-human antibodies, purified or directly conjugated with fluorescein isothiocyanate, phycoerythrin, or allophycocyanin, were used in the flow cytometry analysis: anti-CD105, anti-CD73, anti-CD90, anti-CD29, anti-CD44, anti-CD106, anti-HLADR, anti-CD19, anti-CD11b, anti-CD14, anti-CD34, anti-CD31, anti-CD45 and immunoglobulin (Ig) G/IgM isotype controls (all from BD Biosciences, San Jose, CA). For fluorescence measurements only, data from 10,000 single cell events were collected using a standard FACScalibur™ flow cytometer (Immunocytometry Systems/Becton Dickinson, San Jose, CA). Data were analyzed using CELLQuest™ (Becton Dickinson).

### miRNA microarray analysis, miRNA-Gene-network and miRNA-Gene-ontology (GO) network analysis

Ten samples of dMSCs, five from women with normal pregnancies (control group, N1-N5) and five from patients with PE (P1-P5) were assayed using human miRNA microarray kit version 16.0 (Agilent Technologies, Santa Clara, CA) purchased from CapitalBio Corporation (Beijing, China). Total RNA, including miRNAs, was extracted using Trizol reagent (Invitrogen) according to the manufacturer’s instructions. The concentration of RNA was measured using a SmartSpec™ Plus spectrophotometer (Bio-Rad, Hercules, CA), and the purity of RNA was checked by Agilent 2100 Bioanalyzer (the value of A260/A280 was between 1.9 and 2.0), and the quality of RNA was confirmed by agarose gel electrophoresis. For each miRNA, multiple probes were spotted on the array, and the mean intensity of these probes was calculated to represent the expression value of the miRNAs. In addition, multiple spots were included as negative controls. For each sample, 100 ng total RNA was hybridized with the miRNA array and further processed in accordance with the manufacturer’s instructions. Only those miRNAs with significant (p < 0.05) differential expression of ≥ 2.0-fold changes were reported. The scanned images were processed using the Sanger Center miRBase version 16.0. The miRNA-Gene-network was constructed based on the interactions of miRNAs and genes in Sanger miRNA database. The miRNA gene ontology (GO) network was constructed based on the relationships of significant GO categories and genes/miRNAs.

### Quantitative reverse transcription-polymerase chain reaction analysis

Total RNA was purified using miRNA isolation kit (Ambion, Austin, TX) to enrich the small RNA fraction. The expression of miRNAs was determined by SYBR Green assays (Bio-Rad, Hercules, CA). SYBR Green qPCR SuperMix-UDG was purchased from Invitrogen. Quantitative polymerase chain reaction (qPCR) was performed using an Applied Bio- Systems 7500 Fast system. All experiments were performed in triplicate. The level of miRNA expression was calculated based on the PCR cycle number (Ct), and the relative gene expression level was determined using the ^ΔΔ^Ct method. All primers used are listed in Table 2.

**Table 2:** Primer information

**miRNA name**	**Forward primer(5′-3′)**	**Reverse primer (5′-3′)**
miR-136-FP	ACACTCCAGCTGGGACTCCATTTGTTTTG	CTCAACTGGTGTCGTGGAGTCGGCAATTCAGTTGAGTCCATCAT
miR-495-RP	ACACTCCAGCTGGGAAACAAACATGGTG	CTCAACTGGTGTCGTGGAGTCGGCAATTCAGTTGAGAAGAAGTG
miR-494-RP	ACACTCCAGCTGGGTGAAACATACACGG	CTCAACTGGTGTCGTGGAGTCGGCAATTCAGTTGAGGAGGTTTC
miR-16-RP	ACACTCCAGCTGGGTAGCAGCACGTAAA	CTCAACTGGTGTCGTGGAGTCGGCAATTCAGTTGAGCGCCAATA
miR-29b-RP	ACACTCCAGCTGGGTAGCACCATTTTGAAA	CTCAACTGGTGTCGTGGAGTCGGCAATTCAGTTGAGAACACTGA
miR-140-RP	ACACTCCAGCTGGGCAGTGGTTTTACCC	CTCAACTGGTGTCGTGGAGTCGGCAATTCAGTTGAGCTACCATA
miR-30a-RP	ACACTCCAGCTGGGTGTAAACATCCTCG	CTCAACTGGTGTCGTGGAGTCGGCAATTCAGTTGAGCTTCCAGT
miR-100-RP	ACACTCCAGCTGGGAACCCGTAGATCCG	CTCAACTGGTGTCGTGGAGTCGGCAATTCAGTTGAGCACAAGTT
miR-221-RP	ACACTCCAGCTGGGAGCTACATTGTCTGC	CTCAACTGGTGTCGTGGAGTCGGCAATTCAGTTGAGGAAACCA
miR-1207-RP	ACACTCCAGCTGGGTGGCAGGGAGGCT	CTCAACTGGTGTCGTGGAGTCGGCAATTCAGTTGAGCCCCTCCC
U6snRNA	CTCGCTTCGGCAGCACA	AACGCTTCACGAATTTGCGT
URP	TGGTGTCGTGGAGTCG	
SOCS3	CCTGCGCCTCAAGACCTTC	GTCACTGCGCTCCAGTAGAA
PPP2R2A	CATACCAGGTGCATGAATACCTC	GGGTTATGTCTCGCTTTGTGTTT
GAPDH	GGAGCGAGATCCCTCCAAAAT	GGCTGTTGTCATACTTCTCATGG

### Luciferase assays

Cells were plated in 24-well plates at a density of 1.5 × 10^4^ cells per well and each well received 250 ng pGL3-luciferase reporter and 5 ng Renilla luciferase reporter. The cells were harvested using Promega’s Passive Lysis buffer after the indicated treatment. Luciferase and Renilla luciferase activities were determined using Promega’s Dual Luciferase assay in a Plate Chameleon luminometer (BioScan, Washington DC). Firefly luciferase was normalized by Renilla luciferase to correct for transfection efficiency. Fold induction was determined by dividing the averaged normalized values from each treatment by the control value for each transfection condition within that experiment. Values were averaged from multiple experiments as indicated in the figure legends.

### Data analysis

The acquired array images were analyzed with Agilent Feature Extraction software (version 10.7.3.1). Quality normalization and subsequent data processing were performed with the Agilent GeneSpring GX software package (v11.5.1). Differentially expressed miRNAs were identified through Fold Change filtering and hierarchically clustered by the Agilent GeneSpring GX software (version 11.5.1). Statistical analysis was performed using unpaired Student’s t-test, using Graphpad Prism 5 Demo software (GraphPad software, San Diego, CA). A p < 0.05 was considered to be statistically significant.

## Results

### Identification of dMSCs derived from patients with PE and healthy donors

**Figure 1:**
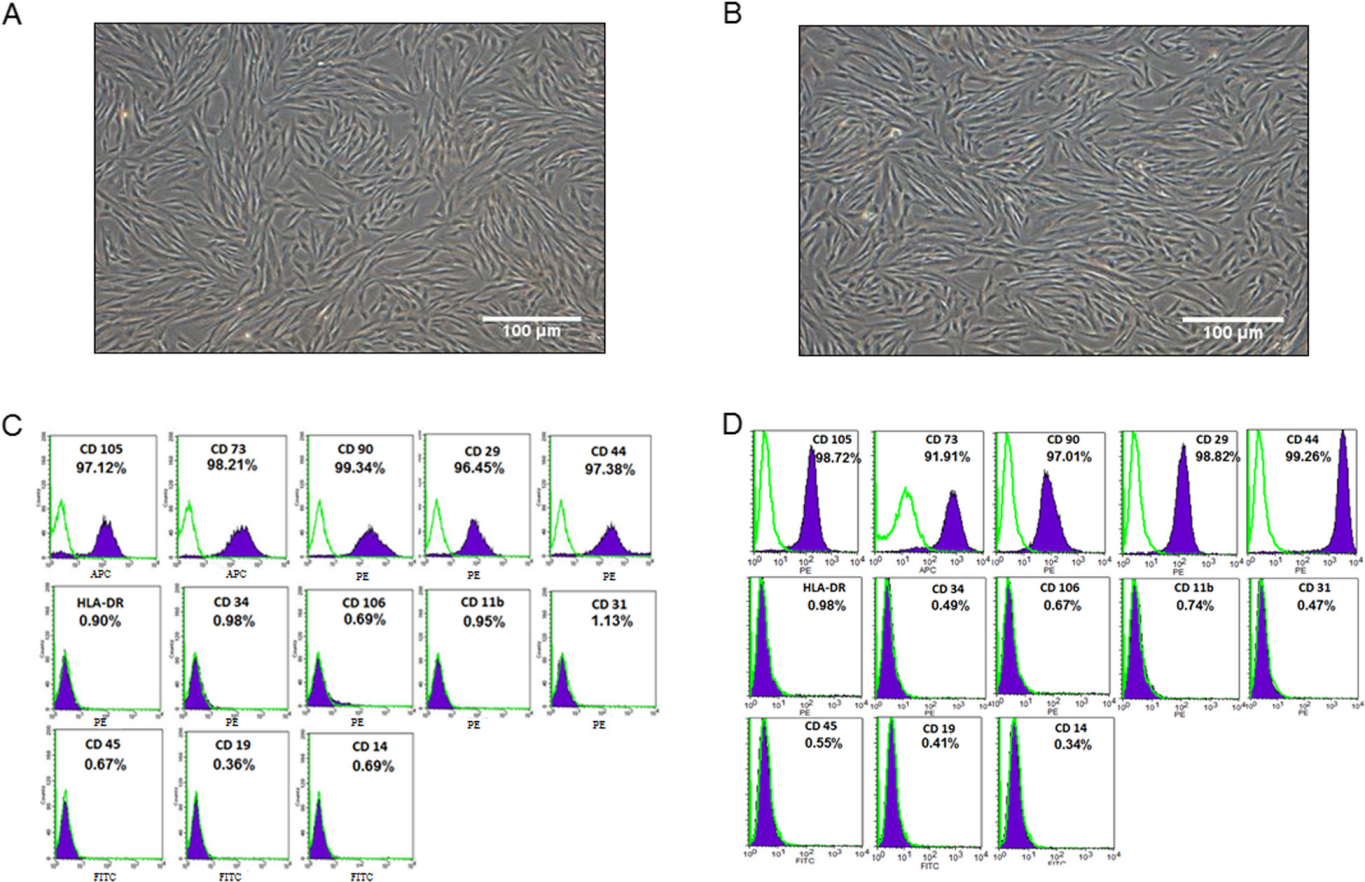
**Identification of dMSCs derived from patients with PE and healthy donors. A**: Morphology of dMSCs from healthy pregnant women within 1 week of culture; **B**: Morphology of dMSCs from patients with PE within 1 week of culture; **C**: Flow cytometric characterization of dMSCs isolated from healthy pregnant woman during passage 3. Expression of surface antigens CD105, CD73, CD90, CD29, CD44, HLA-DR, CD19, CD11b, CD106, CD45, CD14, CD34, and CD31 was detected using flow cytometry. The percentage of each positive marker is shown. The percentages are shown as mean ± SE from five healthy pregnant women; **D**: Flow cytometric characterization of dMSCs isolated from patient with PE during passage 3. Expression of surface antigens CD105, CD73, CD90, CD29, CD44, HLA-DR, CD19, CD11b, CD106, CD45, CD14, CD34, and CD31 was detected using flow cytometry. The percentages are shown as mean ± SE from five patients with PE. dMSC, decidua-derived mesenchymal stem cell; PE, pre-eclampsia; SE, standard error.

The decidua tissues were collected from patients with PE and age-matched normotensive controls. Then the dMSCs were isolated and cultured as described in Methods. After two cell passages, the cells formed a monolayer of homogeneous bipolar spindle-like cells with a whirlpool-like array (Figure 1A and B). Furthermore, after three cell passages, the adherent cells were symmetric with phenotypic surface antigens. The dMSCs shared most of their phenotypes with bone marrow-derived MSCs as reported previously [34]–[36], including positivity for CD29, CD44, CD90, CD105 (SH2) and CD73 (SH3), and negativity for CD19, CD11b, CD14, CD34, CD106, CD45 and CD31 (endothelial cell marker), and HLA-DR (Figure 1C and D). The results showed that dMSCs from patients with PE and healthy controls maintained similar cell morphology and phenotype (Figure 1).

### miRNA expression profiles and validation of microarray data by qPCR

Using the Agilent human miRNA microarray kit version 16.0, consisting of 940 miRNA probes, corresponding to the Sanger Center miRBase version 16.0, miRNA expression profiling was performed in the dMSCs derived from the healthy women with normal pregnancies (N1-N5) and women with PE (P1-P5). The comparison between these populations is expected to reveal some underlying differences regarding the activation of gene expression programs related to maintenance, proliferation and function of stem cells, providing important insights into the pathophysiology of PE.

**Figure 2:**
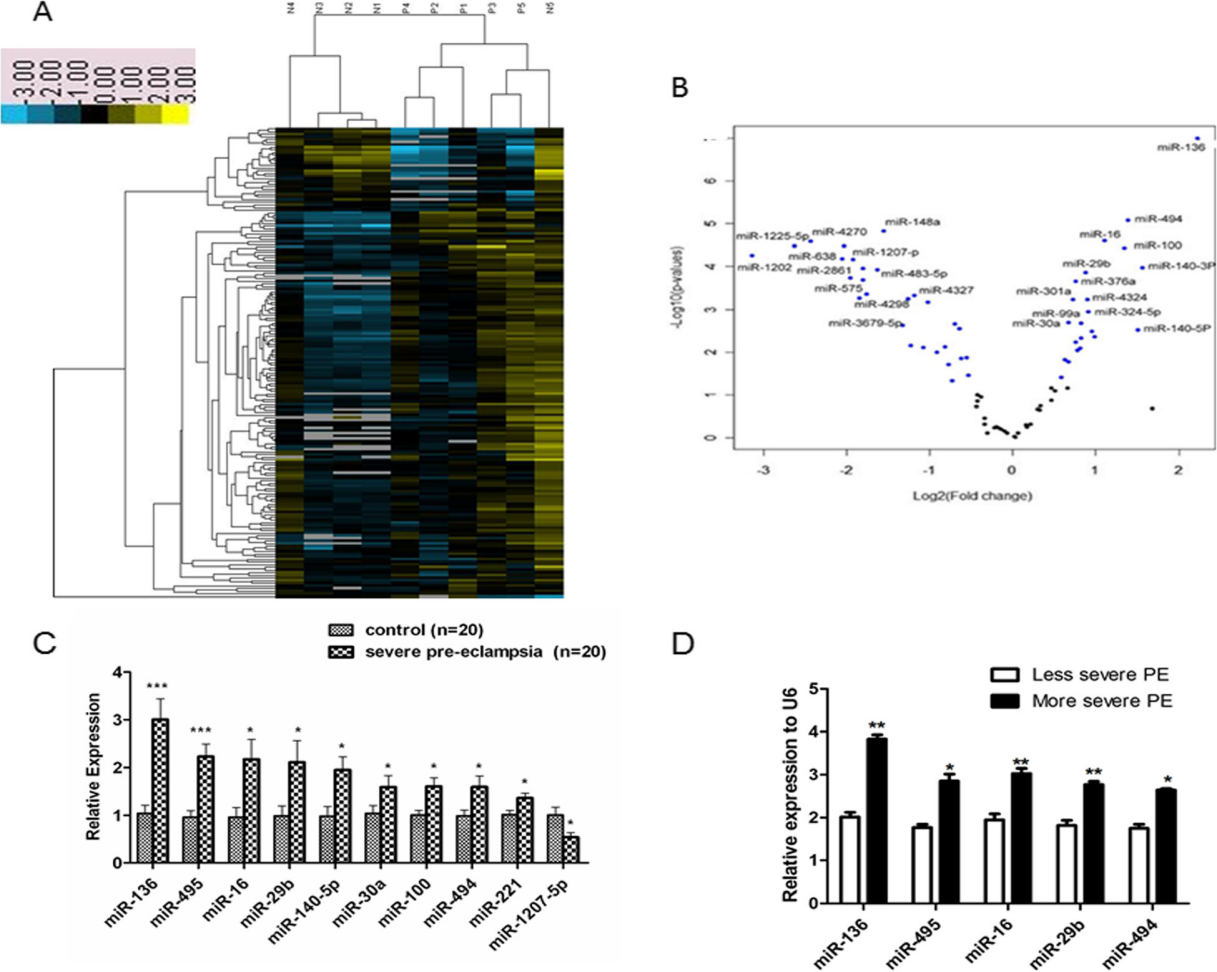
**miRNA expression profiles and validation of microarray data by qRT-PCR analysis. A**: Hierarchical cluster analysis of differentially expressed miRNAs in placenta from patients with PE. miRNAs in decidua from women with PE (P1, P2, P3, P4 and P5) and healthy pregnant women (N1, N2, N3, N4 and N5). Each row represents a miRNA and each column represents a sample pair of decidua patients with PE and healthy women with normal pregnancies. The color legend indicates high expression (yellow), low expression (blue) or no change in expression (Black). The grey segments represent the amount of miRNA expression is very low or no hybridization signal is detected. The miRNA data were clustered based on their similarities in expression among these cohorts; **B**: Differentially expressed miRNAs in patients with PE (n = 5) and healthy pregnant women (n = 5). Average log2 expression ratio of all differentially expressed miRNAs (p < 0.01). Blue dots represent the miRNAs with significant (p < 0.05) differential expression of ≥ 2.0-fold changes. Black dots represent the miRNAs with differential expression of >1.0-fold and <2.0-fold changes (p > 0.05); **C**: qPCR was used to analyze the expression of miR-136, miR-495, miR-16, miR-29b, miR-140-5p, miR-30a, miR-100, miR-494, miR-221, and miR-1207-5p in dMSCs from patients with PE. **D**: qPCR was used to analyze the expression of miR-136, miR-495, miR-16, miR-29b, and miR-494 in dMSCs from patients with more severe PE (proteinuria, >2108 mg/24 h, systolic blood pressure, >160.4 mmHg; diastolic blood pressure, >110.4 mmHg) and less severe PE (proteinuria, <2108 mg/24 h, systolic blood pressure, <160.4 mmHg; diastolic blood pressure, <110.4 mmHg). Data indicate relative expression following normalization. Values are means ± SE (*p < 0.05; ***p < 0.01). dMSC, decidua-derived mesenchymal stem cell; miRNA, microRNA; PE, pre-eclampsia; qPCR, quantitative polymerase chain reaction.

To obtain an overview of the similarities and differences in their miRNA expression profiles, we performed an unsupervised clustering analysis of our expression datasets after normalizing miRNA Ct values across samples with the quantile method and filtering out miRNAs with low variation across all samples. The hierarchical cluster of the genes with > 2-fold changes between healthy women with normal pregnancies and patients with PE is shown in Figure 2A. The biological replicates of each cell type were read together, demonstrating the robustness of this dataset. Remarkably, the miRNA expression profile of dMCSs from patients with PE is different from that of healthy women with normal pregnancies.

As shown in Figure 2B, compared with healthy women with normal pregnancies, miR-136, miR-494, miR-16, miR-100, miR-29b, miR-140-3p, miR-376a, miR-301a, miR-4324, miR-324-5p, miR-99a, miR-30a and miR-140-5p were significantly increased, while miR-148a, miR-4270, miR-1225-5p, miR-1207, miR-638, miR-1202, miR-2861, miR-483-5p, miR-575, miR-4327, miR-4298 and miR-3679-5p were decreased in dMSCs from patients with PE. Further validation of aberrant miRNAs was determined using qPCR analysis in dMSCs from 20 patients with PE and 20 healthy women with normal pregnancies. The nine most up-regulated miRNAs (miR-136, miR-495, miR-16, miR-29b, miR-140-5p, miR-30a, miR-100, miR-494, and miR-221) and one down-regulated miRNA (miR-1207-5p) were identified. As shown in Figure 2C, miR-136, miR-495, miR-16, miR-29b, miR-140-5p, miR-30a, miR-100, miR-494 and miR-221 were increased and miR-1207-5p was decreased in dMSCs from patients with PE. The results were consistent with the microarray analysis.

Furthermore, the patients with PE were divided into more severe PE (proteinuria, >2108 mg/24 h, systolic blood pressure, >160.4 mmHg; diastolic blood pressure, >110.4 mmHg) and less severe PE (proteinuria, <2108 mg/24 h, systolic blood pressure, <160.4 mmHg; diastolic blood pressure, <110.4 mmHg). Then the expression of miR-136, miR-495, miR-16, miR-29b and miR-494 was detected in these two groups using qPCR. As shown in Figure 2D, miR-136, miR-495, miR-16, miR-29b and miR-494 were expressed more in patients with more severe PE than those in patients with less severe PE. Moreover, the relationship between differentially expressed miRNAs and pathogenesis of PE was analyzed. As listed in Additional file 1: Table S1, miR-16, miR-30a, miR-29b, miR-100, miR-214, miR-148a, and miR-483-5p involved the regulation of angiogenesis; miR-494, miR-140-5p, miR-16, miR-301a, miR-30a, miR-221, and miR-132 played an important role in the regulation of inflammation signaling and function of immune cells; miR-140-3p, miR-140-5p, miR-29b, miR-16, and miR-31regulated the differentiation of dMSCs.

### Differentially expressed miRNA-Gene-network and miRNA-GO-network analysis

To explore the relationship between miRNAs and properties of genes, the miRNA network was constructed based on the relationship between significant GOs, genes, and miRNAs. The miRNA-Gene-network was constructed based on the interactions of miRNAs and genes in the Sanger miRNA database. The miRNA-GO-network was constructed based on the relationships between significant GO categories and genes/miRNAs. In these networks, the degree represents the contribution of an individual miRNA or GO category to adjacent miRNAs or GO categories.

**Figure 3:**
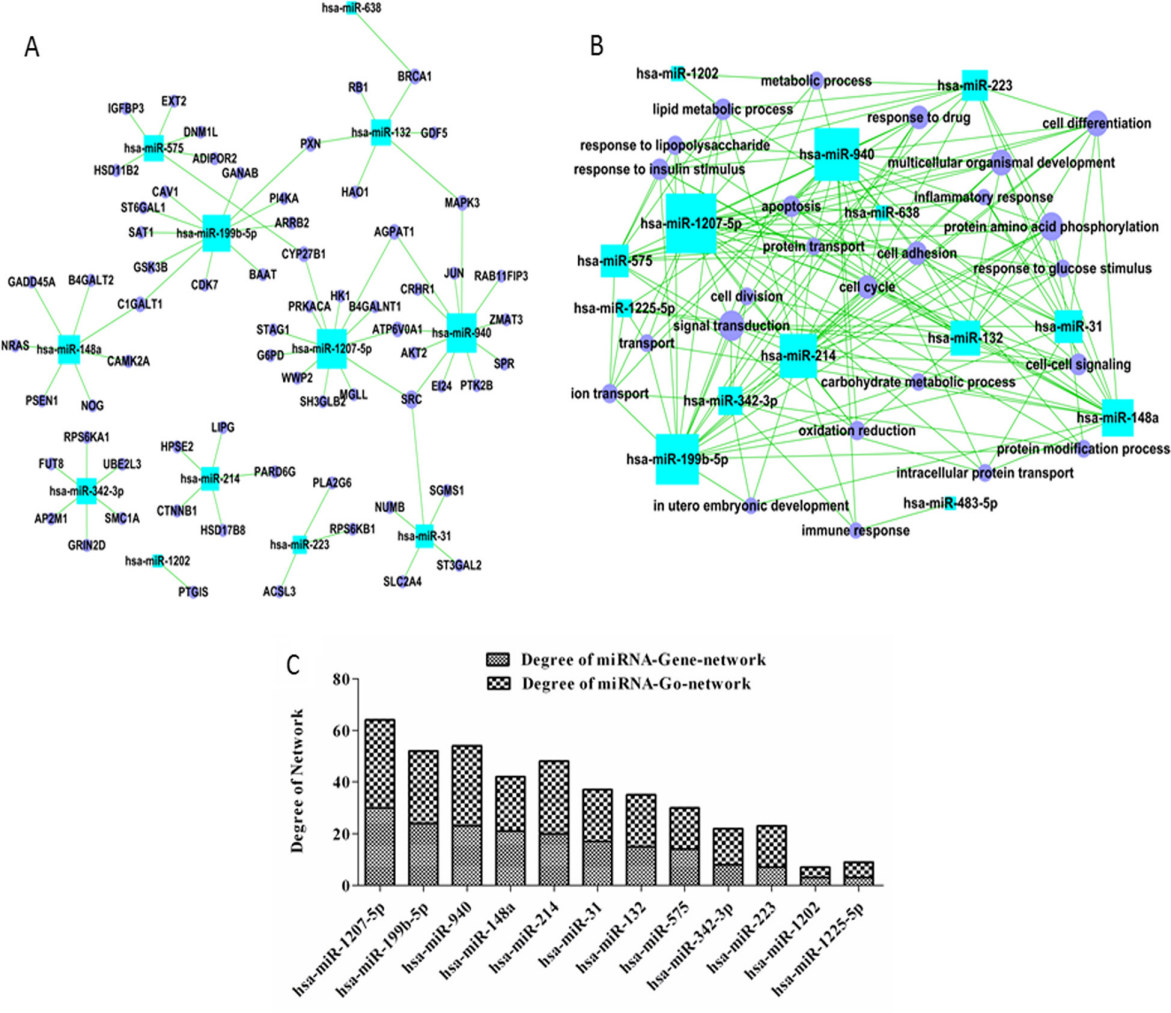
**Differentially expressed miRNA-Gene-network and miRNA-GO-network analysis. A**: The miRNA-Gene-network was constructed based on the interactions of down-regulated miRNAs and genes in Sanger miRNA database; **B**: The miRNA-GO-network was constructed based on the relationships of significant GO categories and down-regulated miRNAs; **C**: The network degree of low-level expressed miRNAs. In these networks, the term “degree” represents the contribution of an individual miRNA or GO category to adjacent genes or GO categories. GO, gene ontology; miRNA, microRNA.

It was reported previously [37] that miR-16 showed the highest number of connections in the up-regulated miRNAs. Other miRNAs also showed higher degrees, including miR-30a, miR-29b, miR-301a, miR-495, miR-494, miR-221, miR-377, and miR-10b. In decreased miRNA-Gene-network and miRNA-GO-network (Figure 3A and 3B), it was found that miR-1207-5p showed the highest contribution to adjacent miRNAs or GO categories in the down-regulated miRNAs (Figure 3C). As shown by Additional file 1: Tables S2 and S3, miR-1207-5p regulated 30 genes and 34 GOs. Other miRNAs including miR-199b-5p (24 genes and 28 GOs), miR-940 (23 genes and 21 GOs), miR-148a (21 genes and 21 GOs) and miR-214 (20 genes and 28 GOs) also showed a higher number of connections with adjacent miRNAs or GO categories (Figure 3C, Additional file 1: Tables S2 and S3).

### Analysis of the signaling pathways regulated by differentially expressed miRNAs

Furthermore, the significantly enriched signaling pathways, which regulated by the differentially expressed miRNAs, were identified. The top 15 enriched signaling pathways for up-regulated and down-regulated expression of miRNAs were shown in Additional file 1: Figure S1A and S1B, respectively. It revealed that the pathways associated with cell proliferation, angiogenesis, and immune functions were highly regulated by differentially expressed miRNAs, including Wnt signaling pathway, mitogen-activated protein kinase (MAPK) signaling pathway, transforming growth factor (TGF) beta signaling pathway, T cell receptor signaling pathway, and B cell receptor signaling pathway, etc. (Additional file 1: Figure S1A and S1B). Moreover, the significantly enriched GOs regulated by differentially expressed miRNAs were analyzed. The degree represents the number of miRNAs that regulate the same GO. As shown in Table 3, angiogenesis, response to hypoxia, apoptosis, TGF beta receptor signaling pathway, cell migration, and immune response were significantly regulated by up-regulated miRNAs in patients with PE. In these GOs, angiogenesis and response to hypoxia were regulated by 15 miRNAs and 14 miRNAs, respectively. Moreover, cell differentiation, cell cycle, apoptosis, aging, and response to lipopolysaccharide were mainly regulated by down-regulated miRNAs in patients with PE (Table 3).

**Table 3:** Target gene signaling pathways analysis

**go_name**	**Degree**
Increased miRNAs in patients with PE	
Angiogenesis	15
Response to hypoxia	14
Apoptosis	13
Transforming growth factor beta receptor signaling pathway	12
Cell migration	11
Immune response	11
Decreased miRNAs in patients with PE	
Cell differentiation	9
Cell cycle	8
Apoptosis	7
Aging	6
Response to lipopolysaccharide	6

Degree represents the number of miRNAs related to a GO.

### Detection of VEGFA, IDO, SOCS3 and PPP2R2A expression in dMSCs

Vascular endothelial growth factor A (VEGFA) is a key angiogenesis factor. Indoleamine 2,3-dioxygenase (IDO) and suppression of cytokine signaling 3 (SOCS3) play an important role in immunoloregulation of MSC. Serine/threonine protein phosphatase 2A 55 kDa regulatory subunit B α isoform (PPP2R2A) negatively regulates extracellular signal-regulated kinase (ERK) pathway [38],[39], which is involved in differentiation of MSCs [40],[41].

**Figure 4:**
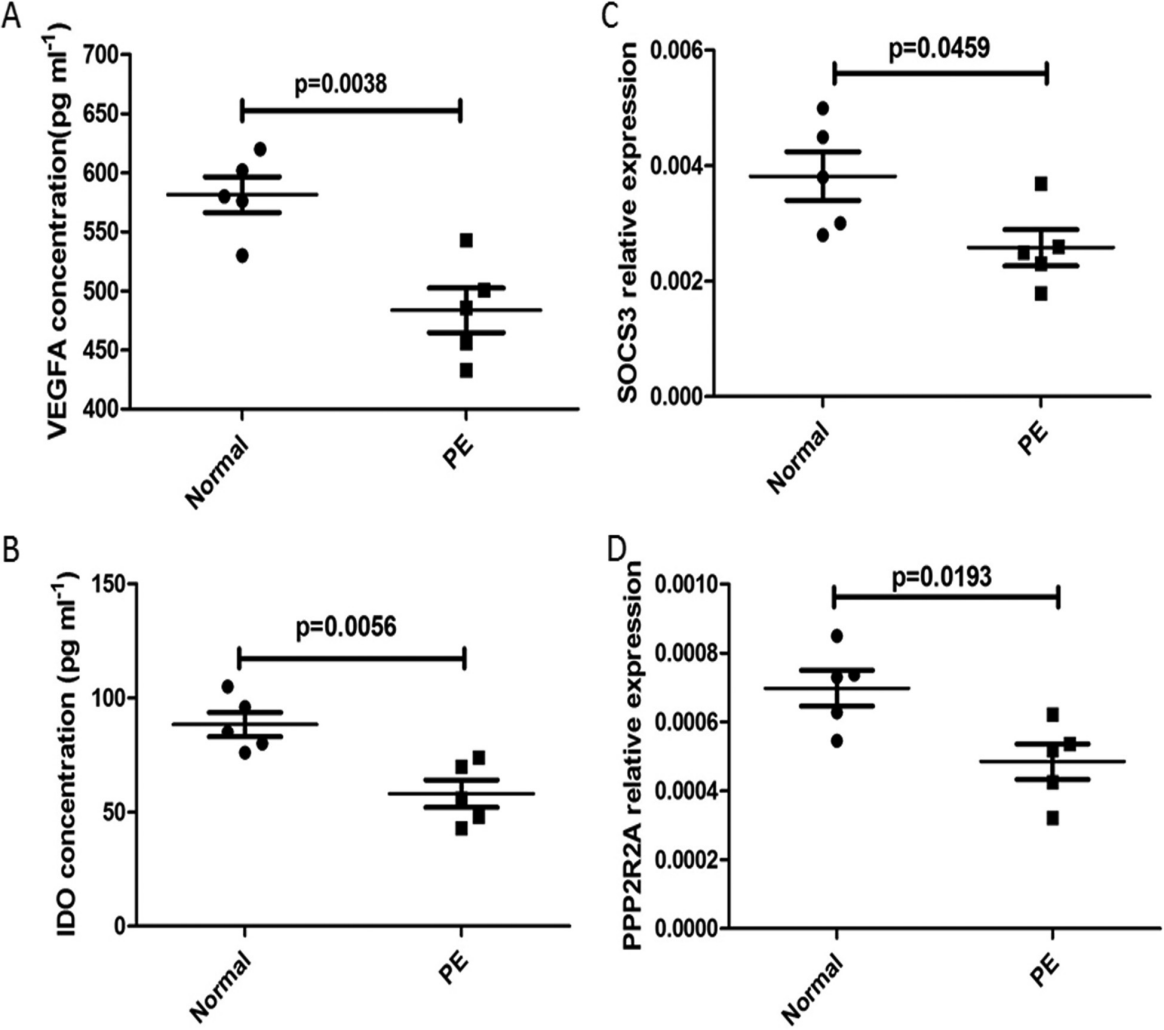
**The expression of miRNA-targeted genes in dMSCs from patients with PE and healthy pregnant women. A**: The serum was isolated from five healthy pregnant women and five patients with PE, and concentration of VEGFA in serum was assessed using ELISA. **B**: The serum was isolated from five healthy pregnant women and five patients with PE, and concentration of IDO in serum was assessed using ELISA. **C**: dMSCs were isolated from five healthy pregnant women and five patients with PE as described in Materials and Methods. Then the expression of SOCS3 was detected by qPCR. **D**: dMSCs were isolated from five healthy pregnant women and five patients with PE as described in Materials and Methods. Then the expression of PPP2R2A was detected by qPCR. dMSC, decidua-derived mesenchymal stem cell; ELISA, enzyme-linked immunosorbent assay; IDO, indoleamine 2,3-dioxygenase; miRNA, microRNA; PE, pre-eclampsia; PPP2R2A, serine/threonine protein phosphatase 2A 55 kDa regulatory subunit B α isoform; qPCR, quantitative polymerase chain reaction; SOCS3, suppression of cytokine signaling 3; VEGFR, vascular endothelial growth factor.

Interestingly, VEGFA was predicted to be a putative target of miR-16 and miR-29b by the miRNA-Gene-network analysis and other target prediction programs [miRanda (http://diana.cslab.ece.ntua.gr/microT/), TargetScan (http://www.targetscan.org/) and PicTar (http://pictar.mdc-berlin.de/) algorithms). With same methods, it was predicted that IDO was a target of miR-494 and SOCS3 was a target of miR-495. In addition, it was reported that PPP2R2A is the target gene of miR-136 [38]. To test whether these changes in miRNA expression were accompanied by changes in corresponding genes, the expression levels of VEGFA, IDO, SOCS3, and PPP2R2A in dMSCs were determined. As shown in Figure 4A-4D, VEGFA, IDO, SOCS3 and PPP2R2A were all decreased in dMSCs from patients with PE compared with healthy pregnant women.

### miR-16 targets VEGFA and miR-136 targets PPP2R2A in dMSCs

**Figure 5:**
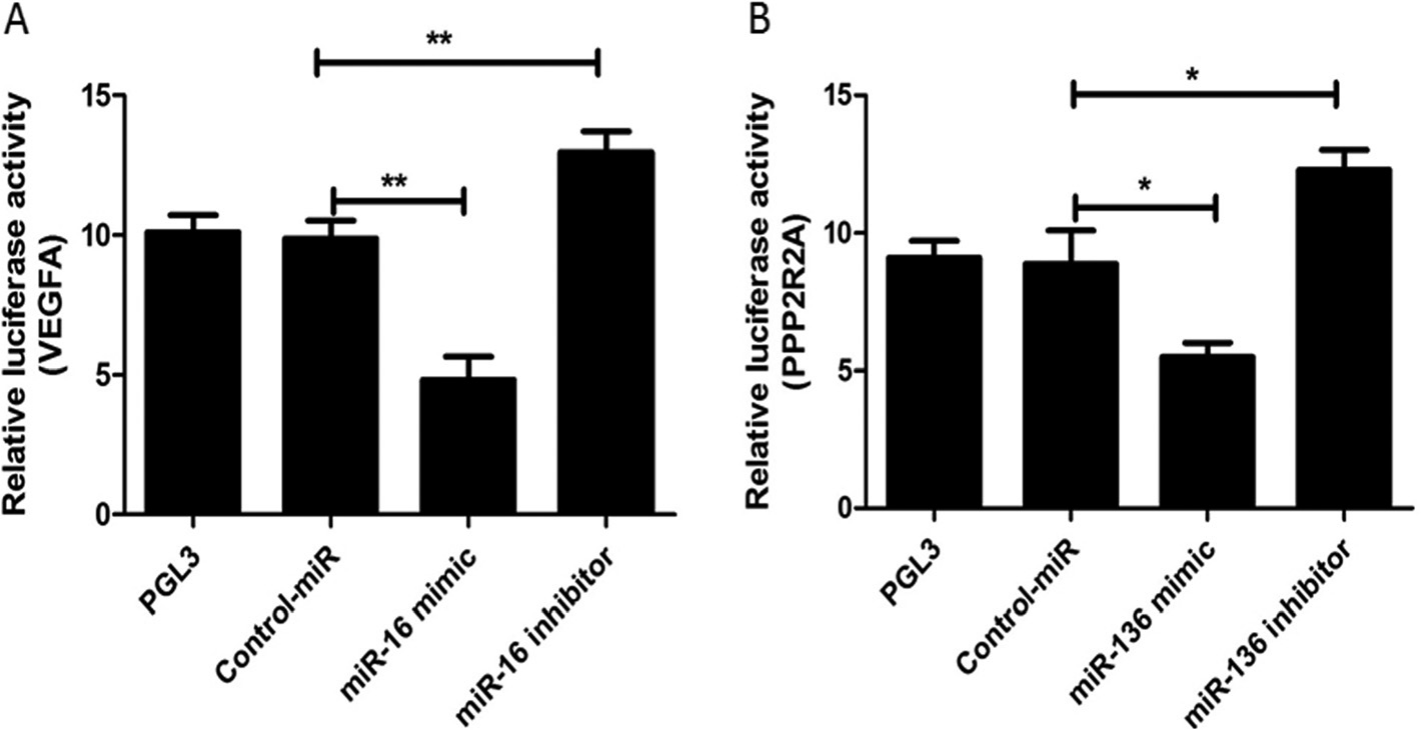
**Validation of miRNA target. A**: The luciferase activity of dMSCs was measured after co-transfection with the indicated VEGFA 3′ UTR constructs and miR-16 or its inhibitor for 24 h. **B**: The luciferase activity of dMSCs was measured after co-transfection with the indicated PPP2R2A 3′ UTR constructs and miR-136 or its inhibitor for 24 h. The results are shown as mean ± SE from three representative independent experiments. *p < 0.05, **P < 0.01 compared with miR-negative. dMSC, decidua-derived mesenchymal stem cell; miRNA, microRNA; PPP2R2A, serine/threonine protein phosphatase 2A 55 kDa regulatory subunit B α isoform; SE, standard error, UTR, untranslated region; VEGFR, vascular endothelial growth factor.

miR-16 showed the highest number of connections in the differentially expressed miRNAs. More importantly, miR-16 was predicted to target VEGFA, which is an important candidate for the pathogenesis of PE. In addition, miR-136 was found to be highly up-regulated in the validation experiment. Therefore, miR-16 and miR-136 were chosen to validate the predicted target gene in dMSCs. The expression of a luciferase reporter gene fused to 3′ untranslated region (UTR) of VEGFA after transfection with miR-16 mimic and its inhibitor into dMSCs was detected. The results showed that miR-16 significantly suppressed the activity of luciferase, which could be reversed by further introduction of miR-16 inhibitor in dMSCs (Figure 5A). Similarly, miR-136 significantly suppressed the expression of a luciferase reporter gene fused to 3′ UTR of PPP2R2A, which could be reversed by further introduction of miR-136 inhibitor in dMSCs (Figure 5B). These results further indicate that differentially expressed miRNAs may be involved in the pathogenesis of PE.

## Discussion

Previous studies indicated that PE may be a pregnancy-induced autoimmune disease, and the imbalanced immune system in the maternal–fetal interface may be one cause of PE [1],[3],[4]. Moreover, abnormality in placental vascular remodeling is also a likely pathogenesis of PE [9],[10]. These are the two main theories for explaining the development of PE [42]. MSCs are considered to be immune-privileged and shown to exert a strong inhibitory effect on other immune cells [14]–[17], and they have the ability to promote endogenous angiogenesis and neurogenesis through a variety of secreted factors [21]. The maternal–fetal interface is an important source of MSCs [22]–[24]. Therefore, investigation of immune-modulatory, pro-angiogenic and anti-inflammatory properties of MSCs at the maternal–fetal interface may open new perspectives into the understanding of PE [5].

In the present study, miRNA expression profiles showed differentially expressed miRNAs in dMSCs between healthy pregnant women and patients with PE. Differential miRNA expressions were defined as a statistically significant difference with a ≥2-fold change. Significantly up-regulated 21 miRNAs and down-regulated 28 miRNAs are present in patients with PE vs healthy pregnant women. Nine top up-regulated miRNAs including miR-136, miR-495, miR-16, miR-29b, miR-140-5p, miR-30a, miR-100, miR-494, and miR-221 and one down-regulated miRNA, miR-1207-5p, were confirmed by qPCR. A previous study had shown that miR-16 inhibits the proliferation and angiogenesis-regulating potential of dMSC [37]. miR-29b contributes to PE through its effects on apoptosis, invasion and angiogenesis of trophoblast cells [43]. Moreover, another up-regulated miRNA, miR-181a, was proved to regulate immune balance by inhibiting proliferation and immunosuppressive properties of MSCs [44]. Interestingly, it was found that the levels of miR-136, miR-495, miR-16, miR-29b and miR-494 were more in patients with more severe PE (proteinuria, >2108 mg/24 h; systolic blood pressure, >160.4 mm Hg; diastolic blood pressure, >110.4 mm Hg) than those in patients with less severe PE (proteinuria, <2108 mg/24 h; systolic blood pressure, <160.4 mm Hg; diastolic blood pressure, <110.4 mm Hg).

Target gene signaling pathway analysis showed that angiogenesis and response to hypoxia were significantly regulated by differentially expressed miRNAs. Abnormality in placental vascular remodeling is a likely pathogenesis of PE [9],[10]. In particular, an imbalance in circulating proangiogenic and antiangiogenic factors released by the hypoxic placenta has gained currency as a critical link between placental dysfunction and several maternal manifestations of PE, particularly endothelial dysfunction and proteinuria [45]. Besides, it was reported that miR-16 and miR-29b, which could regulate angiogenesis [37],[43], and another three up-regulated miRNAs, miR-140, miR-30a, and miR-100, were also associated with angiogenesis [46]–[48].

GO analysis also showed that miRNAs involve many immune response signaling pathways in patients with PE including the MAPK signaling pathway, TGF beta signaling pathway, T-cell receptor signaling pathway, and B-cell receptor signaling pathway, etc. In a previous study, it was found that miR-181a is an immune-regulating factor [44]. miR-181a can regulate the TGF-beta signaling pathway by targeting TGFBR1 and TGFBRAP1 in dMSCs. In addition, miR-181a can also enhance secretion of interleukin (IL)-6 and IDO by activating p38 and JNK signaling pathways in dMSCs [44]. Moreover, it was reported that miR-16 modulates nuclear factor-kappaB-regulated transactivation of IL-8 gene [49]. miRNA-29 is involved in the adaptive immune system and immune-modulation [50],[51]. miR-140 regulates TGF-β1/Smad3 pathway [52]. miRNA-30a and miRNA-221 regulate B cells and mast cells, respectively [53],[54].

## Conclusions

miRNA expression profiles of patients with PE are significantly different from that of healthy pregnant women. Altered miRNAs lead to excessive activation or inactivation of signaling pathways in dMSCs. These aberrant changes result in abnormality of immune-modulatory, pro-angiogenic and anti-inflammatory properties of dMSCs. Our study further indicates that differentially expressed miRNAs may be involved in the pathogenesis of PE.

## Abbreviations

MSC: Mesenchymal stem cell

dMSC: Decidua-derived mesenchymal stem cell

PE: Pre-eclampsia

miRNAs: microRNAs

TGF beta: Transforming growth factor beta

MAPK: Mitogen-activated protein kinase

## Competing interests

The authors declared that they have no competing interests.

## Authors’ contributions

ZGF, HYY and HYL designed and carried out the experiments and wrote the manuscript. ZX, CSW, MHS, FHY and WZQ discussed the experimental design and results with ZGF and participated in manuscript writing. All authors read and approve the final manuscript.

## Additional file

## Supplementary Material

Additional file 1: Figure S1. Target gene signaling pathways analysis. **A.** The significantly enriched signaling pathways which regulated by up-regulated miRNAs. The top fifteen enriched signaling pathways were showed; **B.** The significantly enriched signaling pathways which regulated by down-regulated miRNAs. The top fifteen enriched signaling pathways were showed. **Table S1.** The functions of differential expressed miRNAs. **Table S2.** Decreased miRNA-Gene-network. **Table S3.** Decreased miRNA-GO-network. Click here for file
