# Supplementary material for: Differential expression of microRNAs in decidua-derived mesenchymal stem cells from patients with pre-eclampsia
Source: J Biomed Sci. 2014 Aug 19;21(1):81. doi: 10.1186/s12929-014-0081-3 (PMC4237795; doi:10.1186/s12929-014-0081-3)
Supplement: Additional file 1: Figure S1. — Target gene signaling pathways analysis. A. The significantly enriched signaling pathways which regulated by up-regulated miRNAs. The top fifteen enriched signaling pathways were showed; B. The significantly enriched signaling pathways which regulated by down-regulated miRNAs. The top fifteen enriched signaling pathways were showed. Table S1. The functions of differential expressed miRNAs. Table S2. Decreased miRNA-Gene-network. Table S3. Decreased miRNA-GO-network. [file s12929-014-0081-3-S1.doc]

**Figure S1**


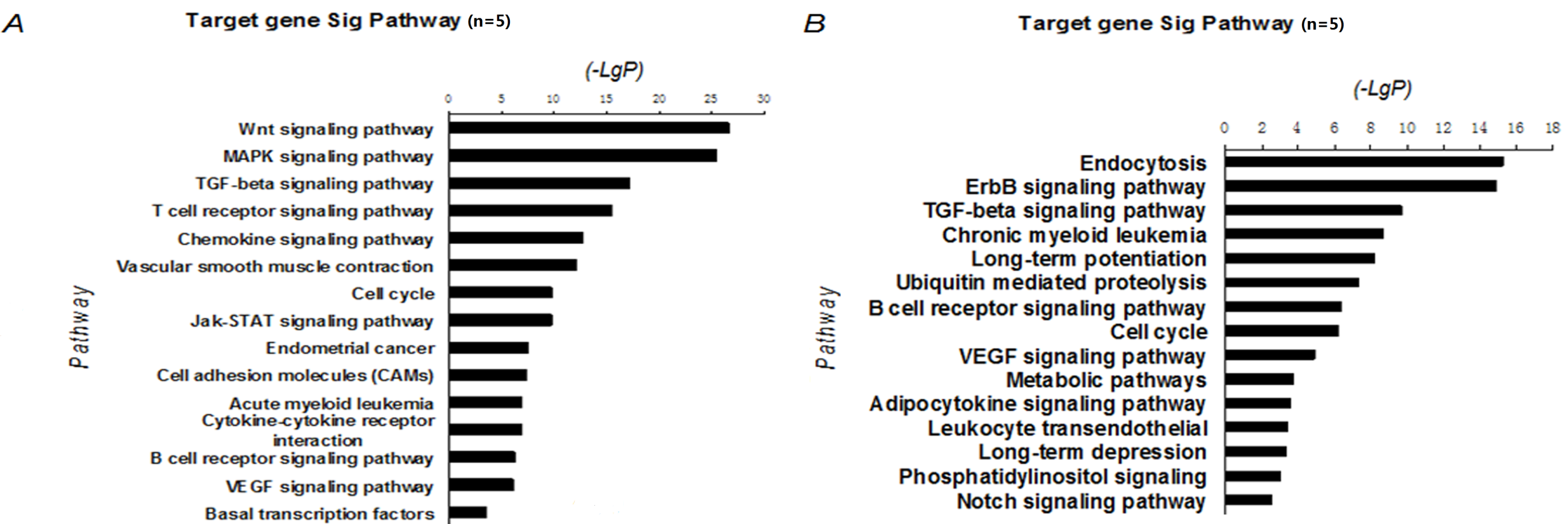


**Figure S1 Target gene signaling pathways analysis.** A, The significantly enriched signaling pathways which regulated by up-regulated miRNAs. The top fifteen enriched signaling pathways were showed; B, The significantly enriched signaling pathways which regulated by down-regulated miRNAs. The top fifteen enriched signaling pathways were showed.

**Table S1 The functions of differential expressed miRNAs**

| **MicroRNA** | **Fold change (P/N)** | **p-value** | | **Function** |
| --- | --- | --- | --- | --- |
| hsa-miR-136 | 4.695 | | 1.00E-07 | Promotes phosphorylation of Erk1/2^[1]^; regulates chondrogenic differentiation of hADSCs^[2]^; pregnancy-associated microRNAs^[3]^. |
| hsa-miR-140-3p | 2.947 | | 0.0001053 | Regulates differentiation of human MSC^[4]^ |
| hsa-miR-494 | 2.83 | | 0.0029885 | Inflammation regulation^[5]^; response to hypoxia^[6]^. |
| hsa-miR-140-5p | 2.607 | | 0.0000083 | Regulate TGFβ pathway^[7]^; obesity related^[8]^; regulates differentiation of MSC^[4]^. |
| hsa-miR-100 | 2.541 | | 0.0000364 | Regulates neovascularization^[9]^; regulates mTOR signaling^[10]^. |
| hsa-miR-29b | 2.144 | | 0.0000248 | Affects apoptosis, invasion and angiogenesis of trophoblast cells^[11]^; immune regulation^[12]^; regulates differentiation of stem cells^[13]^; response to reactive oxygen species^[14]^. |
| hsa-miR-16 | 1.874 | | 0.0011228 | Regulates proliferation and angiogenesis-regulating potential of MSC^[15]^; regulates differentiation and survival of MSC^[16]^; regulates angiogenesis^[17]^;regulates inflammatory signaling^[18]^. |
| hsa-miR-10b | 1.83 | | 0.0001394 | Promotes the migration of MSC^[19]^; regulates TGF-β signaling^[20]^. |
| hsa-miR-376a | 1.776 | | 0.0021417 | Regulation of erythroid differentiation^[21]^. |
| hsa-miR-301a | 1.772 | | 0.0047288 | Regulates NF-κB signaling^[22]^; immune cells regulation^[23]^; affected the cytokine secretion of MSCs^[24]^; regulates Wnt/β-catenin signaling^[25]^. |
| hsa-miR-99a | 1.748 | | 0.0081555 | Regulates mTOR signaling ^[26]^; regulates TGFβ and Wnt signaling^[27]^. |
| hsa-miR-30a | 1.713 | | 0.0090087 | Regulates TGFβ signaling^[28]^; regulates angiogenesis^[29]^; immune cells regulation^[30]^. |
| hsa-miR-376c | 1.696 | | 0.0002228 | Regulates proliferation and invasion of trophoblast cell by regulation TGF-β signaling^[31]^. |
| hsa-miR-127-3p | 1.689 | | 0.0057784 | Regulates TGF-β signaling^[32]^. |
| hsa-miR-590-5p | 1.596 | | 0.0167421 | Regulates Wnt Pathway^[33]^. |
| hsa-miR-221 | 1.592 | | 0.0020338 | Differentially expressed miRNAs in patients with PE^[34]^; regulates differentiation of stem cells^[35]^; immune cells regulation^[36]^. |
| hsa-miR-495 | 1.551 | | 0.015156 | Regulates differentiation of MSC^[37]^. |
| hsa-miR-1915 | 0.689 | | 0.0352405 | Regulates the stemness and repair capacity of renal progenitors ^[38]^. |
| hsa-miR-214 | 0.64 | | 0.0028115 | Regulates angiogenesis^[39]^. |
| hsa-miR-132 | 0.615 | | 0.0021719 | Regulates angiogenesis^[40]^; modulates cholinergic signaling and inflammation^[41]^. |
| hsa-miR-223 | 0.583 | | 0.0193898 | Immune cells regulation^[42]^; regulates embryonic stem cell differentiation^[43]^. |
| hsa-miR-31 | 0.492 | | 0.0006826 | Regulates differentiation of MSC^[44]^. |
| hsa-miR-199b-5p | 0.476 | | 0.0079633 | Regulates erythroid differentiation^[45]^. |
| hsa-miR-148a | 0.34 | | 0.0000149 | Regulates the differentiation of MSC^[46]^; control human MSC phenotype^[64]^; Regulate angiogenesis^[47]^. |
| hsa-miR-483-5p | 0.322 | | 0.0001199 | Controls angiogenesis^[48]^. |
| hsa-miR-4281 | 0.277 | | 0.0005389 | Characterizing the Treg phenotype^[49]^. |
| hsa-miR-1207-5p | 0.264 | | 0.000068 | Involves the pathogenesis of diabetic nephropathy^[50]^. |
| hsa-miR-2861 | 0.258 | | 0.0001808 | Regulate osteoblast differentiation^[51]^. |
| hsa-miR-638 | 0.241 | | 0.0000651 | Predictor of early virological response to interferon treatment in chronic hepatitis B patients^[52]^; play a role in the pathogenesis of lupus nephritis^[53]^. |
| hsa-miR-1225-5p | 0.162 | | 0.0000333 | Regulates the stemness and repair capacity of renal progenitors ^[38]^. |

**Table S2 Decreased miRNA-Gene-network**

| **MicroRNA** | **Degree** |
| --- | --- |
| hsa-miR-1207-5p | 30 |
| hsa-miR-199b-5p | 24 |
| hsa-miR-940 | 23 |
| hsa-miR-148a | 21 |
| hsa-miR-214 | 20 |
| hsa-miR-31 | 17 |
| hsa-miR-132 | 15 |
| hsa-miR-575 | 14 |
| hsa-miR-342-3p | 8 |
| hsa-miR-223 | 7 |
| hsa-miR-1202 | 3 |
| hsa-miR-1225-5p | 3 |
| hsa-miR-483-5p | 1 |
| hsa-miR-572 | 1 |
| hsa-miR-638 | 1 |

Degree represents the contribution of an individual miRNA to adjacent genes.

**Table S3 Decreased miRNA-GO-network**

| **MicroRNA** | **Degree** |
| --- | --- |
| hsa-miR-1207-5p | 34 |
| hsa-miR-940 | 31 |
| hsa-miR-199b-5p | 28 |
| hsa-miR-214 | 28 |
| hsa-miR-148a | 21 |
| hsa-miR-132 | 20 |
| hsa-miR-31 | 20 |
| hsa-miR-223 | 16 |
| hsa-miR-575 | 16 |
| hsa-miR-342-3p | 14 |
| hsa-miR-1225-5p | 6 |
| hsa-miR-1202 | 4 |
| hsa-miR-638 | 3 |
| hsa-miR-483-5p | 1 |
| hsa-miR-572 | 1 |

Degree represents the contribution of an individual miRNA to adjacent GO categories.

**References for Table S1**

1. [Shen S](http://www.ncbi.nlm.nih.gov/pubmed?term=Shen S%5BAuthor%5D&cauthor=true&cauthor_uid=23959478), [Yue H](http://www.ncbi.nlm.nih.gov/pubmed?term=Yue H%5BAuthor%5D&cauthor=true&cauthor_uid=23959478), [Li Y](http://www.ncbi.nlm.nih.gov/pubmed?term=Li Y%5BAuthor%5D&cauthor=true&cauthor_uid=23959478), [Qin J](http://www.ncbi.nlm.nih.gov/pubmed?term=Qin J%5BAuthor%5D&cauthor=true&cauthor_uid=23959478), [Li K](http://www.ncbi.nlm.nih.gov/pubmed?term=Li K%5BAuthor%5D&cauthor=true&cauthor_uid=23959478), [Liu Y](http://www.ncbi.nlm.nih.gov/pubmed?term=Liu Y%5BAuthor%5D&cauthor=true&cauthor_uid=23959478), [Wang J](http://www.ncbi.nlm.nih.gov/pubmed?term=Wang J%5BAuthor%5D&cauthor=true&cauthor_uid=23959478):**Upregulation of miR-136 in human non-small cell lung cancer cells promotes Erk1/2 activation by targeting PPP2R2A**. [*Tumour Biol*](http://www.ncbi.nlm.nih.gov/pubmed/23959478) 2014,**35(1)**:631-40.

2.[Zhang Z](http://www.ncbi.nlm.nih.gov/pubmed?term=Zhang Z%5BAuthor%5D&cauthor=true&cauthor_uid=22947280), [Kang Y](http://www.ncbi.nlm.nih.gov/pubmed?term=Kang Y%5BAuthor%5D&cauthor=true&cauthor_uid=22947280), [Zhang Z](http://www.ncbi.nlm.nih.gov/pubmed?term=Zhang Z%5BAuthor%5D&cauthor=true&cauthor_uid=22947280), [Zhang H](http://www.ncbi.nlm.nih.gov/pubmed?term=Zhang H%5BAuthor%5D&cauthor=true&cauthor_uid=22947280), [Duan X](http://www.ncbi.nlm.nih.gov/pubmed?term=Duan X%5BAuthor%5D&cauthor=true&cauthor_uid=22947280), [Liu J](http://www.ncbi.nlm.nih.gov/pubmed?term=Liu J%5BAuthor%5D&cauthor=true&cauthor_uid=22947280), [Li X](http://www.ncbi.nlm.nih.gov/pubmed?term=Li X%5BAuthor%5D&cauthor=true&cauthor_uid=22947280), [Liao W](http://www.ncbi.nlm.nih.gov/pubmed?term=Liao W%5BAuthor%5D&cauthor=true&cauthor_uid=22947280): **Expression of microRNAs during chondrogenesis of human adipose-derived stem cells**. [*Osteoarthritis Cartilage*](http://www.ncbi.nlm.nih.gov/pubmed/22947280) 2012,**20(12)**:1638-46.

3. [Kotlabova K](http://www.ncbi.nlm.nih.gov/pubmed?term=Kotlabova K%5BAuthor%5D&cauthor=true&cauthor_uid=21513988), [Doucha J](http://www.ncbi.nlm.nih.gov/pubmed?term=Doucha J%5BAuthor%5D&cauthor=true&cauthor_uid=21513988), [Hromadnikova I](http://www.ncbi.nlm.nih.gov/pubmed?term=Hromadnikova I%5BAuthor%5D&cauthor=true&cauthor_uid=21513988): **Placental-specific microRNA in maternal circulation--identification of appropriate pregnancy-associated microRNAs with diagnostic potential**. [*J Reprod Immunol*](http://www.ncbi.nlm.nih.gov/pubmed/21513988) 2011,**89(2)**:185-91.

4. [Karlsen TA](http://www.ncbi.nlm.nih.gov/pubmed?term=Karlsen TA%5BAuthor%5D&cauthor=true&cauthor_uid=24063364), [Jakobsen RB](http://www.ncbi.nlm.nih.gov/pubmed?term=Jakobsen RB%5BAuthor%5D&cauthor=true&cauthor_uid=24063364), [Mikkelsen TS](http://www.ncbi.nlm.nih.gov/pubmed?term=Mikkelsen TS%5BAuthor%5D&cauthor=true&cauthor_uid=24063364), [Brinchmann JE](http://www.ncbi.nlm.nih.gov/pubmed?term=Brinchmann JE%5BAuthor%5D&cauthor=true&cauthor_uid=24063364): **microRNA-140 targets RALA and regulates chondrogenic differentiation of human mesenchymal stem cells by translational enhancement of SOX9 and ACAN**. [*Stem Cells Dev*](http://www.ncbi.nlm.nih.gov/pubmed/24063364) 2014,**23(3)**:290-304.

5. [Lee H](http://www.ncbi.nlm.nih.gov/pubmed?term=Lee H%5BAuthor%5D&cauthor=true&cauthor_uid=24349514), [Jee Y](http://www.ncbi.nlm.nih.gov/pubmed?term=Jee Y%5BAuthor%5D&cauthor=true&cauthor_uid=24349514), [Hong K](http://www.ncbi.nlm.nih.gov/pubmed?term=Hong K%5BAuthor%5D&cauthor=true&cauthor_uid=24349514), [Hwang GS](http://www.ncbi.nlm.nih.gov/pubmed?term=Hwang GS%5BAuthor%5D&cauthor=true&cauthor_uid=24349514), [Chun KH](http://www.ncbi.nlm.nih.gov/pubmed?term=Chun KH%5BAuthor%5D&cauthor=true&cauthor_uid=24349514): **MicroRNA-494, upregulated by tumor necrosis factor-α, desensitizes insulin effect in C2C12 muscle cells**. [*PLoS One*](http://www.ncbi.nlm.nih.gov/pubmed/24349514)2013,**8(12)**:e83471.

6. [Sun G](http://www.ncbi.nlm.nih.gov/pubmed?term=Sun G%5BAuthor%5D&cauthor=true&cauthor_uid=24364919), [Zhou Y](http://www.ncbi.nlm.nih.gov/pubmed?term=Zhou Y%5BAuthor%5D&cauthor=true&cauthor_uid=24364919), [Li H](http://www.ncbi.nlm.nih.gov/pubmed?term=Li H%5BAuthor%5D&cauthor=true&cauthor_uid=24364919), [Guo Y](http://www.ncbi.nlm.nih.gov/pubmed?term=Guo Y%5BAuthor%5D&cauthor=true&cauthor_uid=24364919), [Shan J](http://www.ncbi.nlm.nih.gov/pubmed?term=Shan J%5BAuthor%5D&cauthor=true&cauthor_uid=24364919), [Xia M](http://www.ncbi.nlm.nih.gov/pubmed?term=Xia M%5BAuthor%5D&cauthor=true&cauthor_uid=24364919), [Li Y](http://www.ncbi.nlm.nih.gov/pubmed?term=Li Y%5BAuthor%5D&cauthor=true&cauthor_uid=24364919), [Li S](http://www.ncbi.nlm.nih.gov/pubmed?term=Li S%5BAuthor%5D&cauthor=true&cauthor_uid=24364919), [Long D](http://www.ncbi.nlm.nih.gov/pubmed?term=Long D%5BAuthor%5D&cauthor=true&cauthor_uid=24364919), [Feng L](http://www.ncbi.nlm.nih.gov/pubmed?term=Feng L%5BAuthor%5D&cauthor=true&cauthor_uid=24364919): **Over-expression of microRNA-494 up-regulates hypoxia-inducible factor-1 alpha expression via PI3K/Akt pathway and protects against hypoxia-induced apoptosis**. [*J Biomed Sci*](http://www.ncbi.nlm.nih.gov/pubmed/24364919) 2013,**20**:100.

7. [Yang H](http://www.ncbi.nlm.nih.gov/pubmed?term=Yang H%5BAuthor%5D&cauthor=true&cauthor_uid=23401231), [Fang F](http://www.ncbi.nlm.nih.gov/pubmed?term=Fang F%5BAuthor%5D&cauthor=true&cauthor_uid=23401231), [Chang R](http://www.ncbi.nlm.nih.gov/pubmed?term=Chang R%5BAuthor%5D&cauthor=true&cauthor_uid=23401231), [Yang L](http://www.ncbi.nlm.nih.gov/pubmed?term=Yang L%5BAuthor%5D&cauthor=true&cauthor_uid=23401231): **MicroRNA-140-5p suppresses tumor growth and metastasis by targeting transforming growth factor β receptor 1 and fibroblast growth factor 9 in hepatocellular carcinoma**. [*Hepatology*](http://www.ncbi.nlm.nih.gov/pubmed/23401231) 2013,**58(1)**:205-17.

8. [Ortega FJ](http://www.ncbi.nlm.nih.gov/pubmed?term=Ortega FJ%5BAuthor%5D&cauthor=true&cauthor_uid=23396142), [Mercader JM](http://www.ncbi.nlm.nih.gov/pubmed?term=Mercader JM%5BAuthor%5D&cauthor=true&cauthor_uid=23396142), [Catalán V](http://www.ncbi.nlm.nih.gov/pubmed?term=Catalán V%5BAuthor%5D&cauthor=true&cauthor_uid=23396142), [Moreno-Navarrete JM](http://www.ncbi.nlm.nih.gov/pubmed?term=Moreno-Navarrete JM%5BAuthor%5D&cauthor=true&cauthor_uid=23396142), [Pueyo N](http://www.ncbi.nlm.nih.gov/pubmed?term=Pueyo N%5BAuthor%5D&cauthor=true&cauthor_uid=23396142), [Sabater M](http://www.ncbi.nlm.nih.gov/pubmed?term=Sabater M%5BAuthor%5D&cauthor=true&cauthor_uid=23396142), [Gómez-Ambrosi J](http://www.ncbi.nlm.nih.gov/pubmed?term=Gómez-Ambrosi J%5BAuthor%5D&cauthor=true&cauthor_uid=23396142), [Anglada R](http://www.ncbi.nlm.nih.gov/pubmed?term=Anglada R%5BAuthor%5D&cauthor=true&cauthor_uid=23396142), [Fernández-Formoso JA](http://www.ncbi.nlm.nih.gov/pubmed?term=Fernández-Formoso JA%5BAuthor%5D&cauthor=true&cauthor_uid=23396142), [Ricart W](http://www.ncbi.nlm.nih.gov/pubmed?term=Ricart W%5BAuthor%5D&cauthor=true&cauthor_uid=23396142), [Frühbeck G](http://www.ncbi.nlm.nih.gov/pubmed?term=Frühbeck G%5BAuthor%5D&cauthor=true&cauthor_uid=23396142), [Fernández-Real JM](http://www.ncbi.nlm.nih.gov/pubmed?term=Fernández-Real JM%5BAuthor%5D&cauthor=true&cauthor_uid=23396142):**Targeting the circulating microRNA signature of obesity**. [*Clin Chem*](http://www.ncbi.nlm.nih.gov/pubmed/23396142)2013,**59(5)**:781-92.

9. [Leonhardt F](http://www.ncbi.nlm.nih.gov/pubmed?term=Leonhardt F%5BAuthor%5D&cauthor=true&cauthor_uid=23327924), [Grundmann S](http://www.ncbi.nlm.nih.gov/pubmed?term=Grundmann S%5BAuthor%5D&cauthor=true&cauthor_uid=23327924), [Behe M](http://www.ncbi.nlm.nih.gov/pubmed?term=Behe M%5BAuthor%5D&cauthor=true&cauthor_uid=23327924), [Bluhm F](http://www.ncbi.nlm.nih.gov/pubmed?term=Bluhm F%5BAuthor%5D&cauthor=true&cauthor_uid=23327924), [Dumont RA](http://www.ncbi.nlm.nih.gov/pubmed?term=Dumont RA%5BAuthor%5D&cauthor=true&cauthor_uid=23327924), [Braun F](http://www.ncbi.nlm.nih.gov/pubmed?term=Braun F%5BAuthor%5D&cauthor=true&cauthor_uid=23327924), [Fani M](http://www.ncbi.nlm.nih.gov/pubmed?term=Fani M%5BAuthor%5D&cauthor=true&cauthor_uid=23327924), [Riesner K](http://www.ncbi.nlm.nih.gov/pubmed?term=Riesner K%5BAuthor%5D&cauthor=true&cauthor_uid=23327924), [Prinz G](http://www.ncbi.nlm.nih.gov/pubmed?term=Prinz G%5BAuthor%5D&cauthor=true&cauthor_uid=23327924), [Hechinger AK](http://www.ncbi.nlm.nih.gov/pubmed?term=Hechinger AK%5BAuthor%5D&cauthor=true&cauthor_uid=23327924), [Gerlach UV](http://www.ncbi.nlm.nih.gov/pubmed?term=Gerlach UV%5BAuthor%5D&cauthor=true&cauthor_uid=23327924), [Dierbach H](http://www.ncbi.nlm.nih.gov/pubmed?term=Dierbach H%5BAuthor%5D&cauthor=true&cauthor_uid=23327924), [Penack O](http://www.ncbi.nlm.nih.gov/pubmed?term=Penack O%5BAuthor%5D&cauthor=true&cauthor_uid=23327924), [Schmitt-Gräff A](http://www.ncbi.nlm.nih.gov/pubmed?term=Schmitt-Gräff A%5BAuthor%5D&cauthor=true&cauthor_uid=23327924), [Finke J](http://www.ncbi.nlm.nih.gov/pubmed?term=Finke J%5BAuthor%5D&cauthor=true&cauthor_uid=23327924), [Weber WA](http://www.ncbi.nlm.nih.gov/pubmed?term=Weber WA%5BAuthor%5D&cauthor=true&cauthor_uid=23327924), [Zeiser R](http://www.ncbi.nlm.nih.gov/pubmed?term=Zeiser R%5BAuthor%5D&cauthor=true&cauthor_uid=23327924): **Inflammatory neovascularization during graft-versus-host disease is regulated by αv integrin and miR-100**. [*Blood*](http://www.ncbi.nlm.nih.gov/pubmed/23327924)2013**,121(17)**:3307-18.

10. [Li XJ](http://www.ncbi.nlm.nih.gov/pubmed?term=Li XJ%5BAuthor%5D&cauthor=true&cauthor_uid=24030073), [Luo XQ](http://www.ncbi.nlm.nih.gov/pubmed?term=Luo XQ%5BAuthor%5D&cauthor=true&cauthor_uid=24030073), [Han BW](http://www.ncbi.nlm.nih.gov/pubmed?term=Han BW%5BAuthor%5D&cauthor=true&cauthor_uid=24030073), [Duan FT](http://www.ncbi.nlm.nih.gov/pubmed?term=Duan FT%5BAuthor%5D&cauthor=true&cauthor_uid=24030073), [Wei PP](http://www.ncbi.nlm.nih.gov/pubmed?term=Wei PP%5BAuthor%5D&cauthor=true&cauthor_uid=24030073), [Chen YQ](http://www.ncbi.nlm.nih.gov/pubmed?term=Chen YQ%5BAuthor%5D&cauthor=true&cauthor_uid=24030073): **MicroRNA-100/99a, deregulated in acute lymphoblastic leukaemia, suppress proliferation and promote apoptosis by regulating the FKBP51 and IGF1R/mTOR signalling pathways**. [*Br J Cancer*](http://www.ncbi.nlm.nih.gov/pubmed/24030073) 2013,**109(8)**:2189-98.

11. [Li P](http://www.ncbi.nlm.nih.gov/pubmed?term=Li P%5BAuthor%5D&cauthor=true&cauthor_uid=22716646), [Guo W](http://www.ncbi.nlm.nih.gov/pubmed?term=Guo W%5BAuthor%5D&cauthor=true&cauthor_uid=22716646), [Du L](http://www.ncbi.nlm.nih.gov/pubmed?term=Du L%5BAuthor%5D&cauthor=true&cauthor_uid=22716646), [Zhao J](http://www.ncbi.nlm.nih.gov/pubmed?term=Zhao J%5BAuthor%5D&cauthor=true&cauthor_uid=22716646), [Wang Y](http://www.ncbi.nlm.nih.gov/pubmed?term=Wang Y%5BAuthor%5D&cauthor=true&cauthor_uid=22716646), [Liu L](http://www.ncbi.nlm.nih.gov/pubmed?term=Liu L%5BAuthor%5D&cauthor=true&cauthor_uid=22716646), [Hu Y](http://www.ncbi.nlm.nih.gov/pubmed?term=Hu Y%5BAuthor%5D&cauthor=true&cauthor_uid=22716646), [Hou Y](http://www.ncbi.nlm.nih.gov/pubmed?term=Hou Y%5BAuthor%5D&cauthor=true&cauthor_uid=22716646): **microRNA-29b contributes to pre-eclampsia through its effects on apoptosis, invasion and angiogenesis of trophoblast cells**. [*Clin Sci (Lond)*](http://www.ncbi.nlm.nih.gov/pubmed/22716646) 2013,**124(1)**:27-40.

12. [Kirigin FF](http://www.ncbi.nlm.nih.gov/pubmed?term=Kirigin FF%5BAuthor%5D&cauthor=true&cauthor_uid=22379031), [Lindstedt K](http://www.ncbi.nlm.nih.gov/pubmed?term=Lindstedt K%5BAuthor%5D&cauthor=true&cauthor_uid=22379031), [Sellars M](http://www.ncbi.nlm.nih.gov/pubmed?term=Sellars M%5BAuthor%5D&cauthor=true&cauthor_uid=22379031), [Ciofani M](http://www.ncbi.nlm.nih.gov/pubmed?term=Ciofani M%5BAuthor%5D&cauthor=true&cauthor_uid=22379031), [Low SL](http://www.ncbi.nlm.nih.gov/pubmed?term=Low SL%5BAuthor%5D&cauthor=true&cauthor_uid=22379031), [Jones L](http://www.ncbi.nlm.nih.gov/pubmed?term=Jones L%5BAuthor%5D&cauthor=true&cauthor_uid=22379031), [Bell F](http://www.ncbi.nlm.nih.gov/pubmed?term=Bell F%5BAuthor%5D&cauthor=true&cauthor_uid=22379031), [Pauli F](http://www.ncbi.nlm.nih.gov/pubmed?term=Pauli F%5BAuthor%5D&cauthor=true&cauthor_uid=22379031), [Bonneau R](http://www.ncbi.nlm.nih.gov/pubmed?term=Bonneau R%5BAuthor%5D&cauthor=true&cauthor_uid=22379031), [Myers RM](http://www.ncbi.nlm.nih.gov/pubmed?term=Myers RM%5BAuthor%5D&cauthor=true&cauthor_uid=22379031), [Littman DR](http://www.ncbi.nlm.nih.gov/pubmed?term=Littman DR%5BAuthor%5D&cauthor=true&cauthor_uid=22379031), [Chong MM](http://www.ncbi.nlm.nih.gov/pubmed?term=Chong MM%5BAuthor%5D&cauthor=true&cauthor_uid=22379031): **Dynamic microRNA gene transcription and processing during T cell development**. [*J Immunol*](http://www.ncbi.nlm.nih.gov/pubmed/22379031) 2012,**188(7)**:3257-67.

13. [Suh JS](http://www.ncbi.nlm.nih.gov/pubmed?term=Suh JS%5BAuthor%5D&cauthor=true&cauthor_uid=23478036), [Lee JY](http://www.ncbi.nlm.nih.gov/pubmed?term=Lee JY%5BAuthor%5D&cauthor=true&cauthor_uid=23478036), [Choi YS](http://www.ncbi.nlm.nih.gov/pubmed?term=Choi YS%5BAuthor%5D&cauthor=true&cauthor_uid=23478036), [Chong PC](http://www.ncbi.nlm.nih.gov/pubmed?term=Chong PC%5BAuthor%5D&cauthor=true&cauthor_uid=23478036), [Park YJ](http://www.ncbi.nlm.nih.gov/pubmed?term=Park YJ%5BAuthor%5D&cauthor=true&cauthor_uid=23478036): **Peptide-mediated intracellular delivery of miRNA-29b for osteogenic stem cell differentiation**. [*Biomaterials*](http://www.ncbi.nlm.nih.gov/pubmed/23478036) 2013,**34(17)**:4347-59.

14. [Xu Z](http://www.ncbi.nlm.nih.gov/pubmed?term=Xu Z%5BAuthor%5D&cauthor=true&cauthor_uid=24657470), [Zhang L](http://www.ncbi.nlm.nih.gov/pubmed?term=Zhang L%5BAuthor%5D&cauthor=true&cauthor_uid=24657470), [Fei X](http://www.ncbi.nlm.nih.gov/pubmed?term=Fei X%5BAuthor%5D&cauthor=true&cauthor_uid=24657470), [Yi X](http://www.ncbi.nlm.nih.gov/pubmed?term=Yi X%5BAuthor%5D&cauthor=true&cauthor_uid=24657470), [Li W](http://www.ncbi.nlm.nih.gov/pubmed?term=Li W%5BAuthor%5D&cauthor=true&cauthor_uid=24657470), [Wang Q](http://www.ncbi.nlm.nih.gov/pubmed?term=Wang Q%5BAuthor%5D&cauthor=true&cauthor_uid=24657470): **The miR-29b-Sirt1 axis regulates self-renewal of mouse embryonic stem cells in response to reactive oxygen species**. *Cell Signal*, 2014,**26(7)**:1500-1505.

15. [Wang Y](http://www.ncbi.nlm.nih.gov/pubmed?term=Wang Y%5BAuthor%5D&cauthor=true&cauthor_uid=23083510), [Fan H](http://www.ncbi.nlm.nih.gov/pubmed?term=Fan H%5BAuthor%5D&cauthor=true&cauthor_uid=23083510), [Zhao G](http://www.ncbi.nlm.nih.gov/pubmed?term=Zhao G%5BAuthor%5D&cauthor=true&cauthor_uid=23083510), [Liu D](http://www.ncbi.nlm.nih.gov/pubmed?term=Liu D%5BAuthor%5D&cauthor=true&cauthor_uid=23083510), [Du L](http://www.ncbi.nlm.nih.gov/pubmed?term=Du L%5BAuthor%5D&cauthor=true&cauthor_uid=23083510), [Wang Z](http://www.ncbi.nlm.nih.gov/pubmed?term=Wang Z%5BAuthor%5D&cauthor=true&cauthor_uid=23083510), [Hu Y](http://www.ncbi.nlm.nih.gov/pubmed?term=Hu Y%5BAuthor%5D&cauthor=true&cauthor_uid=23083510), [Hou Y](http://www.ncbi.nlm.nih.gov/pubmed?term=Hou Y%5BAuthor%5D&cauthor=true&cauthor_uid=23083510): **miR-16 inhibits the proliferation and angiogenesis-regulating potential of mesenchymal stem cells in severe pre-eclampsia**. *FEBS J* 2012,**279(24)**:4510-24.

16. [Liu JL](http://www.ncbi.nlm.nih.gov/pubmed?term=Liu JL%5BAuthor%5D&cauthor=true&cauthor_uid=22677435), [Jiang L](http://www.ncbi.nlm.nih.gov/pubmed?term=Jiang L%5BAuthor%5D&cauthor=true&cauthor_uid=22677435), [Lin QX](http://www.ncbi.nlm.nih.gov/pubmed?term=Lin QX%5BAuthor%5D&cauthor=true&cauthor_uid=22677435), [Deng CY](http://www.ncbi.nlm.nih.gov/pubmed?term=Deng CY%5BAuthor%5D&cauthor=true&cauthor_uid=22677435), [Mai LP](http://www.ncbi.nlm.nih.gov/pubmed?term=Mai LP%5BAuthor%5D&cauthor=true&cauthor_uid=22677435), [Zhu JN](http://www.ncbi.nlm.nih.gov/pubmed?term=Zhu JN%5BAuthor%5D&cauthor=true&cauthor_uid=22677435), [Li XH](http://www.ncbi.nlm.nih.gov/pubmed?term=Li XH%5BAuthor%5D&cauthor=true&cauthor_uid=22677435), [Yu XY](http://www.ncbi.nlm.nih.gov/pubmed?term=Yu XY%5BAuthor%5D&cauthor=true&cauthor_uid=22677435), [Lin SG](http://www.ncbi.nlm.nih.gov/pubmed?term=Lin SG%5BAuthor%5D&cauthor=true&cauthor_uid=22677435), [Shan ZX](http://www.ncbi.nlm.nih.gov/pubmed?term=Shan ZX%5BAuthor%5D&cauthor=true&cauthor_uid=22677435): **MicroRNA 16 enhances differentiation of human bone marrow mesenchymal stem cells in a cardiac niche toward myogenic phenotypes in vitro**. [*Life Sci*](http://www.ncbi.nlm.nih.gov/pubmed/22677435) 2012,**90(25-26)**:1020-6.

17. [Lee JK](http://www.ncbi.nlm.nih.gov/pubmed?term=Lee JK%5BAuthor%5D&cauthor=true&cauthor_uid=24391924), [Park SR](http://www.ncbi.nlm.nih.gov/pubmed?term=Park SR%5BAuthor%5D&cauthor=true&cauthor_uid=24391924), [Jung BK](http://www.ncbi.nlm.nih.gov/pubmed?term=Jung BK%5BAuthor%5D&cauthor=true&cauthor_uid=24391924), [Jeon YK](http://www.ncbi.nlm.nih.gov/pubmed?term=Jeon YK%5BAuthor%5D&cauthor=true&cauthor_uid=24391924), [Lee YS](http://www.ncbi.nlm.nih.gov/pubmed?term=Lee YS%5BAuthor%5D&cauthor=true&cauthor_uid=24391924), [Kim MK](http://www.ncbi.nlm.nih.gov/pubmed?term=Kim MK%5BAuthor%5D&cauthor=true&cauthor_uid=24391924), [Kim YG](http://www.ncbi.nlm.nih.gov/pubmed?term=Kim YG%5BAuthor%5D&cauthor=true&cauthor_uid=24391924), [Jang JY](http://www.ncbi.nlm.nih.gov/pubmed?term=Jang JY%5BAuthor%5D&cauthor=true&cauthor_uid=24391924), [Kim CW](http://www.ncbi.nlm.nih.gov/pubmed?term=Kim CW%5BAuthor%5D&cauthor=true&cauthor_uid=24391924):**Exosomes derived from mesenchymal stem cells suppress angiogenesis by down-regulating VEGF expression in breast cancer cells**. [*PLoS One*](http://www.ncbi.nlm.nih.gov/pubmed/24391924)2013,**8(12)**:e84256.

18.[Zhou R](http://www.ncbi.nlm.nih.gov/pubmed?term=Zhou R%5BAuthor%5D&cauthor=true&cauthor_uid=22292036), [Li X](http://www.ncbi.nlm.nih.gov/pubmed?term=Li X%5BAuthor%5D&cauthor=true&cauthor_uid=22292036), [Hu G](http://www.ncbi.nlm.nih.gov/pubmed?term=Hu G%5BAuthor%5D&cauthor=true&cauthor_uid=22292036), [Gong AY](http://www.ncbi.nlm.nih.gov/pubmed?term=Gong AY%5BAuthor%5D&cauthor=true&cauthor_uid=22292036), [Drescher KM](http://www.ncbi.nlm.nih.gov/pubmed?term=Drescher KM%5BAuthor%5D&cauthor=true&cauthor_uid=22292036), [Chen XM](http://www.ncbi.nlm.nih.gov/pubmed?term=Chen XM%5BAuthor%5D&cauthor=true&cauthor_uid=22292036): **miR-16 targets transcriptional corepressor SMRT and modulates NF-kappaB-regulated transactivation of interleukin-8 gene**. [*PLoS One*](http://www.ncbi.nlm.nih.gov/pubmed/22292036) 2012,**7(1)**:e30772.

19. [Zhang F](http://www.ncbi.nlm.nih.gov/pubmed?term=Zhang F%5BAuthor%5D&cauthor=true&cauthor_uid=23921523), [Jing S](http://www.ncbi.nlm.nih.gov/pubmed?term=Jing S%5BAuthor%5D&cauthor=true&cauthor_uid=23921523), [Ren T](http://www.ncbi.nlm.nih.gov/pubmed?term=Ren T%5BAuthor%5D&cauthor=true&cauthor_uid=23921523), [Lin J](http://www.ncbi.nlm.nih.gov/pubmed?term=Lin J%5BAuthor%5D&cauthor=true&cauthor_uid=23921523): **MicroRNA-10b promotes the migration of mouse bone marrow-derived mesenchymal stem cells and downregulates the expression of E-cadherin**. [*Mol Med Rep*](http://www.ncbi.nlm.nih.gov/pubmed/23921523) 2013,**8(4)**:1084-8.

20. [Han X](http://www.ncbi.nlm.nih.gov/pubmed?term=Han X%5BAuthor%5D&cauthor=true&cauthor_uid=24457988), [Yan S](http://www.ncbi.nlm.nih.gov/pubmed?term=Yan S%5BAuthor%5D&cauthor=true&cauthor_uid=24457988), [Weijie Z](http://www.ncbi.nlm.nih.gov/pubmed?term=Weijie Z%5BAuthor%5D&cauthor=true&cauthor_uid=24457988), [Feng W](http://www.ncbi.nlm.nih.gov/pubmed?term=Feng W%5BAuthor%5D&cauthor=true&cauthor_uid=24457988), [Liuxing W](http://www.ncbi.nlm.nih.gov/pubmed?term=Liuxing W%5BAuthor%5D&cauthor=true&cauthor_uid=24457988), [Mengquan L](http://www.ncbi.nlm.nih.gov/pubmed?term=Mengquan L%5BAuthor%5D&cauthor=true&cauthor_uid=24457988), [Qingxia F](http://www.ncbi.nlm.nih.gov/pubmed?term=Qingxia F%5BAuthor%5D&cauthor=true&cauthor_uid=24457988): **Critical role of miR-10b in transforming growth factor-β1-induced epithelial-mesenchymal transition in breast cancer.** [*Cancer Gene Ther*](http://www.ncbi.nlm.nih.gov/pubmed/24457988) 2014,21(2):60-7.

21. [Wang F](http://www.ncbi.nlm.nih.gov/pubmed?term=Wang F%5BAuthor%5D&cauthor=true&cauthor_uid=21556037), [Yu J](http://www.ncbi.nlm.nih.gov/pubmed?term=Yu J%5BAuthor%5D&cauthor=true&cauthor_uid=21556037), [Yang GH](http://www.ncbi.nlm.nih.gov/pubmed?term=Yang GH%5BAuthor%5D&cauthor=true&cauthor_uid=21556037), [Wang XS](http://www.ncbi.nlm.nih.gov/pubmed?term=Wang XS%5BAuthor%5D&cauthor=true&cauthor_uid=21556037), [Zhang JW](http://www.ncbi.nlm.nih.gov/pubmed?term=Zhang JW%5BAuthor%5D&cauthor=true&cauthor_uid=21556037): **Regulation of erythroid differentiation by miR-376a and its targets**. [*Cell Res*](http://www.ncbi.nlm.nih.gov/pubmed/21556037) 2011,**21(8)**:1196-209.

22. [Lu Z](http://www.ncbi.nlm.nih.gov/pubmed?term=Lu Z%5BAuthor%5D&cauthor=true&cauthor_uid=21113131), [Li Y](http://www.ncbi.nlm.nih.gov/pubmed?term=Li Y%5BAuthor%5D&cauthor=true&cauthor_uid=21113131), [Takwi A](http://www.ncbi.nlm.nih.gov/pubmed?term=Takwi A%5BAuthor%5D&cauthor=true&cauthor_uid=21113131), [Li B](http://www.ncbi.nlm.nih.gov/pubmed?term=Li B%5BAuthor%5D&cauthor=true&cauthor_uid=21113131), [Zhang J](http://www.ncbi.nlm.nih.gov/pubmed?term=Zhang J%5BAuthor%5D&cauthor=true&cauthor_uid=21113131), [Conklin DJ](http://www.ncbi.nlm.nih.gov/pubmed?term=Conklin DJ%5BAuthor%5D&cauthor=true&cauthor_uid=21113131), [Young KH](http://www.ncbi.nlm.nih.gov/pubmed?term=Young KH%5BAuthor%5D&cauthor=true&cauthor_uid=21113131), [Martin R](http://www.ncbi.nlm.nih.gov/pubmed?term=Martin R%5BAuthor%5D&cauthor=true&cauthor_uid=21113131), [Li Y](http://www.ncbi.nlm.nih.gov/pubmed?term=Li Y%5BAuthor%5D&cauthor=true&cauthor_uid=21113131): **miR-301a as an NF-κB activator in pancreatic cancer cells.** *E*[*MBO J*](http://www.ncbi.nlm.nih.gov/pubmed/21113131)2011,30(1):57-67.

23. [Mycko MP](http://www.ncbi.nlm.nih.gov/pubmed?term=Mycko MP%5BAuthor%5D&cauthor=true&cauthor_uid=22517757), [Cichalewska M](http://www.ncbi.nlm.nih.gov/pubmed?term=Cichalewska M%5BAuthor%5D&cauthor=true&cauthor_uid=22517757), [Machlanska A](http://www.ncbi.nlm.nih.gov/pubmed?term=Machlanska A%5BAuthor%5D&cauthor=true&cauthor_uid=22517757), [Cwiklinska H](http://www.ncbi.nlm.nih.gov/pubmed?term=Cwiklinska H%5BAuthor%5D&cauthor=true&cauthor_uid=22517757), [Mariasiewicz M](http://www.ncbi.nlm.nih.gov/pubmed?term=Mariasiewicz M%5BAuthor%5D&cauthor=true&cauthor_uid=22517757), [Selmaj KW](http://www.ncbi.nlm.nih.gov/pubmed?term=Selmaj KW%5BAuthor%5D&cauthor=true&cauthor_uid=22517757): **MicroRNA-301a regulation of a T-helper 17 immune response controls autoimmune demyelination**. [*Proc Natl Acad Sci U S A*](http://www.ncbi.nlm.nih.gov/pubmed/22517757) 2012,**109(20)**:E1248-57.

24. [Ma F](http://www.ncbi.nlm.nih.gov/pubmed?term=Ma F%5BAuthor%5D&cauthor=true&cauthor_uid=24035358), [Chen D](http://www.ncbi.nlm.nih.gov/pubmed?term=Chen D%5BAuthor%5D&cauthor=true&cauthor_uid=24035358), [Chi Y](http://www.ncbi.nlm.nih.gov/pubmed?term=Chi Y%5BAuthor%5D&cauthor=true&cauthor_uid=24035358), [Chen F](http://www.ncbi.nlm.nih.gov/pubmed?term=Chen F%5BAuthor%5D&cauthor=true&cauthor_uid=24035358), [Li X](http://www.ncbi.nlm.nih.gov/pubmed?term=Li X%5BAuthor%5D&cauthor=true&cauthor_uid=24035358), [Han Z](http://www.ncbi.nlm.nih.gov/pubmed?term=Han Z%5BAuthor%5D&cauthor=true&cauthor_uid=24035358): **The expression and role of miR-301a in human umbilical cord-derived mesenchymal stromal cells**. [*Cytotherapy*](http://www.ncbi.nlm.nih.gov/pubmed/24035358)2013,**15(12)**:1511-6.

25.[Ma F](http://www.ncbi.nlm.nih.gov/pubmed?term=Ma F%5BAuthor%5D&cauthor=true&cauthor_uid=24315818), [Zhang J](http://www.ncbi.nlm.nih.gov/pubmed?term=Zhang J%5BAuthor%5D&cauthor=true&cauthor_uid=24315818), [Zhong L](http://www.ncbi.nlm.nih.gov/pubmed?term=Zhong L%5BAuthor%5D&cauthor=true&cauthor_uid=24315818), [Wang L](http://www.ncbi.nlm.nih.gov/pubmed?term=Wang L%5BAuthor%5D&cauthor=true&cauthor_uid=24315818), [Liu Y](http://www.ncbi.nlm.nih.gov/pubmed?term=Liu Y%5BAuthor%5D&cauthor=true&cauthor_uid=24315818), [Wang Y](http://www.ncbi.nlm.nih.gov/pubmed?term=Wang Y%5BAuthor%5D&cauthor=true&cauthor_uid=24315818), [Peng L](http://www.ncbi.nlm.nih.gov/pubmed?term=Peng L%5BAuthor%5D&cauthor=true&cauthor_uid=24315818), [Guo B](http://www.ncbi.nlm.nih.gov/pubmed?term=Guo B%5BAuthor%5D&cauthor=true&cauthor_uid=24315818): **Upregulated microRNA-301a in breast cancer promotes tumor metastasis by targeting PTEN and activating Wnt/β-catenin signaling**. [*Gene*](http://www.ncbi.nlm.nih.gov/pubmed/24315818) 2014,**535(2)**:191-7.

26. [Wang L](http://www.ncbi.nlm.nih.gov/pubmed?term=Wang L%5BAuthor%5D&cauthor=true&cauthor_uid=24668416), [Chang L](http://www.ncbi.nlm.nih.gov/pubmed?term=Chang L%5BAuthor%5D&cauthor=true&cauthor_uid=24668416), [Li Z](http://www.ncbi.nlm.nih.gov/pubmed?term=Li Z%5BAuthor%5D&cauthor=true&cauthor_uid=24668416), [Gao Q](http://www.ncbi.nlm.nih.gov/pubmed?term=Gao Q%5BAuthor%5D&cauthor=true&cauthor_uid=24668416), [Cai D](http://www.ncbi.nlm.nih.gov/pubmed?term=Cai D%5BAuthor%5D&cauthor=true&cauthor_uid=24668416), [Tian Y](http://www.ncbi.nlm.nih.gov/pubmed?term=Tian Y%5BAuthor%5D&cauthor=true&cauthor_uid=24668416), [Zeng L](http://www.ncbi.nlm.nih.gov/pubmed?term=Zeng L%5BAuthor%5D&cauthor=true&cauthor_uid=24668416), [Li M](http://www.ncbi.nlm.nih.gov/pubmed?term=Li M%5BAuthor%5D&cauthor=true&cauthor_uid=24668416): **miR-99a and -99b inhibit cervical cancer cell proliferation and invasion by targeting mTOR signaling pathway**. [*Med Oncol*](http://www.ncbi.nlm.nih.gov/pubmed/24668416) 2014,**31(5)**:934.

27.[Emmrich S](http://www.ncbi.nlm.nih.gov/pubmed?term=Emmrich S%5BAuthor%5D&cauthor=true&cauthor_uid=24736844), [Rasche M](http://www.ncbi.nlm.nih.gov/pubmed?term=Rasche M%5BAuthor%5D&cauthor=true&cauthor_uid=24736844), [Schöning J](http://www.ncbi.nlm.nih.gov/pubmed?term=Schöning J%5BAuthor%5D&cauthor=true&cauthor_uid=24736844), [Reimer C](http://www.ncbi.nlm.nih.gov/pubmed?term=Reimer C%5BAuthor%5D&cauthor=true&cauthor_uid=24736844), [Keihani S](http://www.ncbi.nlm.nih.gov/pubmed?term=Keihani S%5BAuthor%5D&cauthor=true&cauthor_uid=24736844), [Maroz A](http://www.ncbi.nlm.nih.gov/pubmed?term=Maroz A%5BAuthor%5D&cauthor=true&cauthor_uid=24736844), [Xie Y](http://www.ncbi.nlm.nih.gov/pubmed?term=Xie Y%5BAuthor%5D&cauthor=true&cauthor_uid=24736844), [Li Z](http://www.ncbi.nlm.nih.gov/pubmed?term=Li Z%5BAuthor%5D&cauthor=true&cauthor_uid=24736844), [Schambach A](http://www.ncbi.nlm.nih.gov/pubmed?term=Schambach A%5BAuthor%5D&cauthor=true&cauthor_uid=24736844), [Reinhardt D](http://www.ncbi.nlm.nih.gov/pubmed?term=Reinhardt D%5BAuthor%5D&cauthor=true&cauthor_uid=24736844), [Klusmann JH](http://www.ncbi.nlm.nih.gov/pubmed?term=Klusmann JH%5BAuthor%5D&cauthor=true&cauthor_uid=24736844): **miR-99a/100~125b tricistrons regulate hematopoietic stem and progenitor cell homeostasis by shifting the balance between TGFβ and Wnt signaling.** [*Genes Dev*](http://www.ncbi.nlm.nih.gov/pubmed/24736844) 2014,**28(8)**:858-74.

28. [Zhou Q](http://www.ncbi.nlm.nih.gov/pubmed?term=Zhou Q%5BAuthor%5D&cauthor=true&cauthor_uid=23831330), [Yang M](http://www.ncbi.nlm.nih.gov/pubmed?term=Yang M%5BAuthor%5D&cauthor=true&cauthor_uid=23831330), [Lan H](http://www.ncbi.nlm.nih.gov/pubmed?term=Lan H%5BAuthor%5D&cauthor=true&cauthor_uid=23831330), [Yu X](http://www.ncbi.nlm.nih.gov/pubmed?term=Yu X%5BAuthor%5D&cauthor=true&cauthor_uid=23831330): **miR-30a negatively regulates TGF-β1-induced epithelial-mesenchymal transition and peritoneal fibrosis by targeting Snai1**. [*Am J Pathol*](http://www.ncbi.nlm.nih.gov/pubmed/23831330) 2013,**183(3)**:808-19.

29. [Huang QB](http://www.ncbi.nlm.nih.gov/pubmed?term=Huang QB%5BAuthor%5D&cauthor=true&cauthor_uid=23826258), [Ma X](http://www.ncbi.nlm.nih.gov/pubmed?term=Ma X%5BAuthor%5D&cauthor=true&cauthor_uid=23826258), [Zhang X](http://www.ncbi.nlm.nih.gov/pubmed?term=Zhang X%5BAuthor%5D&cauthor=true&cauthor_uid=23826258), [Liu SW](http://www.ncbi.nlm.nih.gov/pubmed?term=Liu SW%5BAuthor%5D&cauthor=true&cauthor_uid=23826258), [Ai Q](http://www.ncbi.nlm.nih.gov/pubmed?term=Ai Q%5BAuthor%5D&cauthor=true&cauthor_uid=23826258), [Shi TP](http://www.ncbi.nlm.nih.gov/pubmed?term=Shi TP%5BAuthor%5D&cauthor=true&cauthor_uid=23826258), [Zhang Y](http://www.ncbi.nlm.nih.gov/pubmed?term=Zhang Y%5BAuthor%5D&cauthor=true&cauthor_uid=23826258), [Gao Y](http://www.ncbi.nlm.nih.gov/pubmed?term=Gao Y%5BAuthor%5D&cauthor=true&cauthor_uid=23826258), [Fan Y](http://www.ncbi.nlm.nih.gov/pubmed?term=Fan Y%5BAuthor%5D&cauthor=true&cauthor_uid=23826258), [Ni D](http://www.ncbi.nlm.nih.gov/pubmed?term=Ni D%5BAuthor%5D&cauthor=true&cauthor_uid=23826258), [Wang BJ](http://www.ncbi.nlm.nih.gov/pubmed?term=Wang BJ%5BAuthor%5D&cauthor=true&cauthor_uid=23826258), [Li HZ](http://www.ncbi.nlm.nih.gov/pubmed?term=Li HZ%5BAuthor%5D&cauthor=true&cauthor_uid=23826258), [Zheng T](http://www.ncbi.nlm.nih.gov/pubmed?term=Zheng T%5BAuthor%5D&cauthor=true&cauthor_uid=23826258): **Down-Regulated miR-30a in Clear Cell Renal Cell Carcinoma Correlated with Tumor Hematogenous Metastasis by Targeting Angiogenesis-Specific DLL4**. [*PLoS One*](http://www.ncbi.nlm.nih.gov/pubmed/23826258) 2013,**27;8(6)**:e67294.

30. [Liu Y](http://www.ncbi.nlm.nih.gov/pubmed?term=Liu Y%5BAuthor%5D&cauthor=true&cauthor_uid=23450709), [Dong J](http://www.ncbi.nlm.nih.gov/pubmed?term=Dong J%5BAuthor%5D&cauthor=true&cauthor_uid=23450709), [Mu R](http://www.ncbi.nlm.nih.gov/pubmed?term=Mu R%5BAuthor%5D&cauthor=true&cauthor_uid=23450709), [Gao Y](http://www.ncbi.nlm.nih.gov/pubmed?term=Gao Y%5BAuthor%5D&cauthor=true&cauthor_uid=23450709), [Tan X](http://www.ncbi.nlm.nih.gov/pubmed?term=Tan X%5BAuthor%5D&cauthor=true&cauthor_uid=23450709), [Li Y](http://www.ncbi.nlm.nih.gov/pubmed?term=Li Y%5BAuthor%5D&cauthor=true&cauthor_uid=23450709), [Li Z](http://www.ncbi.nlm.nih.gov/pubmed?term=Li Z%5BAuthor%5D&cauthor=true&cauthor_uid=23450709), [Yang G](http://www.ncbi.nlm.nih.gov/pubmed?term=Yang G%5BAuthor%5D&cauthor=true&cauthor_uid=23450709):**MicroRNA-30a promotes B cell hyperactivity in patients with systemic lupus erythematosus by direct interaction with Lyn**. [*Arthritis Rheum*](http://www.ncbi.nlm.nih.gov/pubmed/23450709)2013, **65(6)**:1603-11.

31. [Fu G](http://www.ncbi.nlm.nih.gov/pubmed?term=Fu G%5BAuthor%5D&cauthor=true&cauthor_uid=23424236), [Ye G](http://www.ncbi.nlm.nih.gov/pubmed?term=Ye G%5BAuthor%5D&cauthor=true&cauthor_uid=23424236), [Nadeem L](http://www.ncbi.nlm.nih.gov/pubmed?term=Nadeem L%5BAuthor%5D&cauthor=true&cauthor_uid=23424236), [Ji L](http://www.ncbi.nlm.nih.gov/pubmed?term=Ji L%5BAuthor%5D&cauthor=true&cauthor_uid=23424236), [Manchanda T](http://www.ncbi.nlm.nih.gov/pubmed?term=Manchanda T%5BAuthor%5D&cauthor=true&cauthor_uid=23424236), [Wang Y](http://www.ncbi.nlm.nih.gov/pubmed?term=Wang Y%5BAuthor%5D&cauthor=true&cauthor_uid=23424236), [Zhao Y](http://www.ncbi.nlm.nih.gov/pubmed?term=Zhao Y%5BAuthor%5D&cauthor=true&cauthor_uid=23424236), [Qiao J](http://www.ncbi.nlm.nih.gov/pubmed?term=Qiao J%5BAuthor%5D&cauthor=true&cauthor_uid=23424236), [Wang YL](http://www.ncbi.nlm.nih.gov/pubmed?term=Wang YL%5BAuthor%5D&cauthor=true&cauthor_uid=23424236), [Lye S](http://www.ncbi.nlm.nih.gov/pubmed?term=Lye S%5BAuthor%5D&cauthor=true&cauthor_uid=23424236), [Yang BB](http://www.ncbi.nlm.nih.gov/pubmed?term=Yang BB%5BAuthor%5D&cauthor=true&cauthor_uid=23424236), [Peng C](http://www.ncbi.nlm.nih.gov/pubmed?term=Peng C%5BAuthor%5D&cauthor=true&cauthor_uid=23424236): **MicroRNA-376c impairs transforming growth factor-β and nodal signaling to promote trophoblast cell proliferation and invasion**. [*Hypertension*](http://www.ncbi.nlm.nih.gov/pubmed/23424236) 2013,**61(4)**:864-72.

32. [Jiang H](http://www.ncbi.nlm.nih.gov/pubmed?term=Jiang H%5BAuthor%5D&cauthor=true&cauthor_uid=24517116), [Jin C](http://www.ncbi.nlm.nih.gov/pubmed?term=Jin C%5BAuthor%5D&cauthor=true&cauthor_uid=24517116), [Liu J](http://www.ncbi.nlm.nih.gov/pubmed?term=Liu J%5BAuthor%5D&cauthor=true&cauthor_uid=24517116), [Hua D](http://www.ncbi.nlm.nih.gov/pubmed?term=Hua D%5BAuthor%5D&cauthor=true&cauthor_uid=24517116), [Zhou F](http://www.ncbi.nlm.nih.gov/pubmed?term=Zhou F%5BAuthor%5D&cauthor=true&cauthor_uid=24517116), [Lou X](http://www.ncbi.nlm.nih.gov/pubmed?term=Lou X%5BAuthor%5D&cauthor=true&cauthor_uid=24517116), [Zhao N](http://www.ncbi.nlm.nih.gov/pubmed?term=Zhao N%5BAuthor%5D&cauthor=true&cauthor_uid=24517116), [Lan Q](http://www.ncbi.nlm.nih.gov/pubmed?term=Lan Q%5BAuthor%5D&cauthor=true&cauthor_uid=24517116), [Huang Q](http://www.ncbi.nlm.nih.gov/pubmed?term=Huang Q%5BAuthor%5D&cauthor=true&cauthor_uid=24517116), [Yoon JG](http://www.ncbi.nlm.nih.gov/pubmed?term=Yoon JG%5BAuthor%5D&cauthor=true&cauthor_uid=24517116), [Zheng S](http://www.ncbi.nlm.nih.gov/pubmed?term=Zheng S%5BAuthor%5D&cauthor=true&cauthor_uid=24517116), [Lin B](http://www.ncbi.nlm.nih.gov/pubmed?term=Lin B%5BAuthor%5D&cauthor=true&cauthor_uid=24517116): **Next generation sequencing analysis of miRNAs: MiR-127-3p inhibits glioblastoma proliferation and activates TGF-β signaling by targeting SKI**. [*OMICS*.](http://www.ncbi.nlm.nih.gov/pubmed/24517116) 2014,**8(3)**:196-206.

33. [Shan X](http://www.ncbi.nlm.nih.gov/pubmed?term=Shan X%5BAuthor%5D&cauthor=true&cauthor_uid=23598417), [Miao Y](http://www.ncbi.nlm.nih.gov/pubmed?term=Miao Y%5BAuthor%5D&cauthor=true&cauthor_uid=23598417), [Fan R](http://www.ncbi.nlm.nih.gov/pubmed?term=Fan R%5BAuthor%5D&cauthor=true&cauthor_uid=23598417), [Qian H](http://www.ncbi.nlm.nih.gov/pubmed?term=Qian H%5BAuthor%5D&cauthor=true&cauthor_uid=23598417), [Chen P](http://www.ncbi.nlm.nih.gov/pubmed?term=Chen P%5BAuthor%5D&cauthor=true&cauthor_uid=23598417), [Liu H](http://www.ncbi.nlm.nih.gov/pubmed?term=Liu H%5BAuthor%5D&cauthor=true&cauthor_uid=23598417), [Yan X](http://www.ncbi.nlm.nih.gov/pubmed?term=Yan X%5BAuthor%5D&cauthor=true&cauthor_uid=23598417), [Li J](http://www.ncbi.nlm.nih.gov/pubmed?term=Li J%5BAuthor%5D&cauthor=true&cauthor_uid=23598417), [Zhou F](http://www.ncbi.nlm.nih.gov/pubmed?term=Zhou F%5BAuthor%5D&cauthor=true&cauthor_uid=23598417): **MiR-590-5P Inhibits Growth of HepG2 Cells via Decrease of S100A10 Expression and Inhibition of the Wnt Pathway**. [*Int J Mol Sci*](http://www.ncbi.nlm.nih.gov/pubmed/23598417) 2013,**14(4)**:8556-69.

34. [Li H](http://www.ncbi.nlm.nih.gov/pubmed?term=Li H%5BAuthor%5D&cauthor=true&cauthor_uid=24195082), [Ge Q](http://www.ncbi.nlm.nih.gov/pubmed?term=Ge Q%5BAuthor%5D&cauthor=true&cauthor_uid=24195082), [Guo L](http://www.ncbi.nlm.nih.gov/pubmed?term=Guo L%5BAuthor%5D&cauthor=true&cauthor_uid=24195082), [Lu Z](http://www.ncbi.nlm.nih.gov/pubmed?term=Lu Z%5BAuthor%5D&cauthor=true&cauthor_uid=24195082): **Maternal plasma miRNAs expression in preeclamptic pregnancies.** [*Biomed Res Int*](http://www.ncbi.nlm.nih.gov/pubmed/?term=miR-221+++preeclampsia) 2013,**2013**:970265.

35. [Bakhshandeh B](http://www.ncbi.nlm.nih.gov/pubmed?term=Bakhshandeh B%5BAuthor%5D&cauthor=true&cauthor_uid=22547036), [Hafizi M](http://www.ncbi.nlm.nih.gov/pubmed?term=Hafizi M%5BAuthor%5D&cauthor=true&cauthor_uid=22547036), [Ghaemi N](http://www.ncbi.nlm.nih.gov/pubmed?term=Ghaemi N%5BAuthor%5D&cauthor=true&cauthor_uid=22547036), [Soleimani M](http://www.ncbi.nlm.nih.gov/pubmed?term=Soleimani M%5BAuthor%5D&cauthor=true&cauthor_uid=22547036): **Down-regulation of miRNA-221 triggers osteogenic differentiation in human stem cells.** [*Biotechnol Lett*](http://www.ncbi.nlm.nih.gov/pubmed/22547036)2012,**34(8)**:1579-87.

36. [Knoll M](http://www.ncbi.nlm.nih.gov/pubmed?term=Knoll M%5BAuthor%5D&cauthor=true&cauthor_uid=23716169), [Simmons S](http://www.ncbi.nlm.nih.gov/pubmed?term=Simmons S%5BAuthor%5D&cauthor=true&cauthor_uid=23716169), [Bouquet C](http://www.ncbi.nlm.nih.gov/pubmed?term=Bouquet C%5BAuthor%5D&cauthor=true&cauthor_uid=23716169), [Grün JR](http://www.ncbi.nlm.nih.gov/pubmed?term=Grün JR%5BAuthor%5D&cauthor=true&cauthor_uid=23716169), [Melchers F](http://www.ncbi.nlm.nih.gov/pubmed?term=Melchers F%5BAuthor%5D&cauthor=true&cauthor_uid=23716169): **miR-221 redirects precursor B cells to the BM and regulates their residence**. [*Eur J Immunol*](http://www.ncbi.nlm.nih.gov/pubmed/23716169) 2013, **43(9)**:2497-506.

37. [Lee S](http://www.ncbi.nlm.nih.gov/pubmed?term=Lee S%5BAuthor%5D&cauthor=true&cauthor_uid=24654627), [Yoon DS](http://www.ncbi.nlm.nih.gov/pubmed?term=Yoon DS%5BAuthor%5D&cauthor=true&cauthor_uid=24654627), [Paik S](http://www.ncbi.nlm.nih.gov/pubmed?term=Paik S%5BAuthor%5D&cauthor=true&cauthor_uid=24654627), [Lee KM](http://www.ncbi.nlm.nih.gov/pubmed?term=Lee KM%5BAuthor%5D&cauthor=true&cauthor_uid=24654627), [Jang Y](http://www.ncbi.nlm.nih.gov/pubmed?term=Jang Y%5BAuthor%5D&cauthor=true&cauthor_uid=24654627), [Lee JW](http://www.ncbi.nlm.nih.gov/pubmed?term=Lee JW%5BAuthor%5D&cauthor=true&cauthor_uid=24654627): **MicroRNA-495 Inhibits Chondrogenic Differentiation in Human Mesenchymal Stem Cells by Targeting Sox9**. [*Stem Cells Dev*](http://www.ncbi.nlm.nih.gov/pubmed/24654627) 2014. [Epub ahead of print]

38. [Sallustio F](http://www.ncbi.nlm.nih.gov/pubmed?term=Sallustio F%5BAuthor%5D&cauthor=true&cauthor_uid=23861881), [Serino G](http://www.ncbi.nlm.nih.gov/pubmed?term=Serino G%5BAuthor%5D&cauthor=true&cauthor_uid=23861881), [Costantino V](http://www.ncbi.nlm.nih.gov/pubmed?term=Costantino V%5BAuthor%5D&cauthor=true&cauthor_uid=23861881), [Curci C](http://www.ncbi.nlm.nih.gov/pubmed?term=Curci C%5BAuthor%5D&cauthor=true&cauthor_uid=23861881), [Cox SN](http://www.ncbi.nlm.nih.gov/pubmed?term=Cox SN%5BAuthor%5D&cauthor=true&cauthor_uid=23861881), [De Palma G](http://www.ncbi.nlm.nih.gov/pubmed?term=De Palma G%5BAuthor%5D&cauthor=true&cauthor_uid=23861881), [Schena FP](http://www.ncbi.nlm.nih.gov/pubmed?term=Schena FP%5BAuthor%5D&cauthor=true&cauthor_uid=23861881): **miR-1915 and miR-1225-5p regulate the expression of CD133, PAX2 and TLR2 in adult renal progenitor cells.** [*PLoS One*](http://www.ncbi.nlm.nih.gov/pubmed/23861881) 2013,**8(7)**:e68296.

39. [van Mil A](http://www.ncbi.nlm.nih.gov/pubmed?term=van Mil A%5BAuthor%5D&cauthor=true&cauthor_uid=22227154), [Grundmann S](http://www.ncbi.nlm.nih.gov/pubmed?term=Grundmann S%5BAuthor%5D&cauthor=true&cauthor_uid=22227154), [Goumans MJ](http://www.ncbi.nlm.nih.gov/pubmed?term=Goumans MJ%5BAuthor%5D&cauthor=true&cauthor_uid=22227154), [Lei Z](http://www.ncbi.nlm.nih.gov/pubmed?term=Lei Z%5BAuthor%5D&cauthor=true&cauthor_uid=22227154), [Oerlemans MI](http://www.ncbi.nlm.nih.gov/pubmed?term=Oerlemans MI%5BAuthor%5D&cauthor=true&cauthor_uid=22227154), [Jaksani S](http://www.ncbi.nlm.nih.gov/pubmed?term=Jaksani S%5BAuthor%5D&cauthor=true&cauthor_uid=22227154), [Doevendans PA](http://www.ncbi.nlm.nih.gov/pubmed?term=Doevendans PA%5BAuthor%5D&cauthor=true&cauthor_uid=22227154), [Sluijter JP](http://www.ncbi.nlm.nih.gov/pubmed?term=Sluijter JP%5BAuthor%5D&cauthor=true&cauthor_uid=22227154):**MicroRNA-214 inhibits angiogenesis by targeting Quaking and reducing angiogenic growth factor release**. [*Cardiovasc Res.*](http://www.ncbi.nlm.nih.gov/pubmed/22227154) 2012,**93(4)**:655-65.

40. [Mulik S](http://www.ncbi.nlm.nih.gov/pubmed?term=Mulik S%5BAuthor%5D&cauthor=true&cauthor_uid=22659469), [Xu J](http://www.ncbi.nlm.nih.gov/pubmed?term=Xu J%5BAuthor%5D&cauthor=true&cauthor_uid=22659469), [Reddy PB](http://www.ncbi.nlm.nih.gov/pubmed?term=Reddy PB%5BAuthor%5D&cauthor=true&cauthor_uid=22659469), [Rajasagi NK](http://www.ncbi.nlm.nih.gov/pubmed?term=Rajasagi NK%5BAuthor%5D&cauthor=true&cauthor_uid=22659469), [Gimenez F](http://www.ncbi.nlm.nih.gov/pubmed?term=Gimenez F%5BAuthor%5D&cauthor=true&cauthor_uid=22659469), [Sharma S](http://www.ncbi.nlm.nih.gov/pubmed?term=Sharma S%5BAuthor%5D&cauthor=true&cauthor_uid=22659469), [Lu PY](http://www.ncbi.nlm.nih.gov/pubmed?term=Lu PY%5BAuthor%5D&cauthor=true&cauthor_uid=22659469), [Rouse BT](http://www.ncbi.nlm.nih.gov/pubmed?term=Rouse BT%5BAuthor%5D&cauthor=true&cauthor_uid=22659469): **Role of miR-132 in angiogenesis after ocular infection with herpes simplex virus.** *Am J Pathol* 2012,**181(2)**:525-34.

41. [Hanieh H](http://www.ncbi.nlm.nih.gov/pubmed?term=Hanieh H%5BAuthor%5D&cauthor=true&cauthor_uid=23780851), [Alzahrani A](http://www.ncbi.nlm.nih.gov/pubmed?term=Alzahrani A%5BAuthor%5D&cauthor=true&cauthor_uid=23780851): **MicroRNA-132 suppresses autoimmune encephalomyelitis by inducing cholinergic anti-inflammation: a new Ahr-based exploration**. [*Eur J Immunol*](http://www.ncbi.nlm.nih.gov/pubmed/23780851) 2013,**43(10)**:2771-82.

42. [McGirt LY](http://www.ncbi.nlm.nih.gov/pubmed?term=McGirt LY%5BAuthor%5D&cauthor=true&cauthor_uid=24304814), [Adams CM](http://www.ncbi.nlm.nih.gov/pubmed?term=Adams CM%5BAuthor%5D&cauthor=true&cauthor_uid=24304814), [Baerenwald DA](http://www.ncbi.nlm.nih.gov/pubmed?term=Baerenwald DA%5BAuthor%5D&cauthor=true&cauthor_uid=24304814), [Zwerner JP](http://www.ncbi.nlm.nih.gov/pubmed?term=Zwerner JP%5BAuthor%5D&cauthor=true&cauthor_uid=24304814), [Zic JA](http://www.ncbi.nlm.nih.gov/pubmed?term=Zic JA%5BAuthor%5D&cauthor=true&cauthor_uid=24304814), [Eischen CM](http://www.ncbi.nlm.nih.gov/pubmed?term=Eischen CM%5BAuthor%5D&cauthor=true&cauthor_uid=24304814): **miR-223 Regulates Cell Growth and Targets Proto-Oncogenes in Mycosis Fungoides/Cutaneous T-Cell Lymphoma**. *J Invest Dermatol* 2014,**134(4)**:1101-7.

43. [Yu YH](http://www.ncbi.nlm.nih.gov/pubmed?term=Yu YH%5BAuthor%5D&cauthor=true&cauthor_uid=24250812), [Zhang L](http://www.ncbi.nlm.nih.gov/pubmed?term=Zhang L%5BAuthor%5D&cauthor=true&cauthor_uid=24250812), [Wu DS](http://www.ncbi.nlm.nih.gov/pubmed?term=Wu DS%5BAuthor%5D&cauthor=true&cauthor_uid=24250812), [Zhang Z](http://www.ncbi.nlm.nih.gov/pubmed?term=Zhang Z%5BAuthor%5D&cauthor=true&cauthor_uid=24250812), [Huang FF](http://www.ncbi.nlm.nih.gov/pubmed?term=Huang FF%5BAuthor%5D&cauthor=true&cauthor_uid=24250812), [Zhang J](http://www.ncbi.nlm.nih.gov/pubmed?term=Zhang J%5BAuthor%5D&cauthor=true&cauthor_uid=24250812), [Chen XP](http://www.ncbi.nlm.nih.gov/pubmed?term=Chen XP%5BAuthor%5D&cauthor=true&cauthor_uid=24250812), [Liang DS](http://www.ncbi.nlm.nih.gov/pubmed?term=Liang DS%5BAuthor%5D&cauthor=true&cauthor_uid=24250812), [Zeng H](http://www.ncbi.nlm.nih.gov/pubmed?term=Zeng H%5BAuthor%5D&cauthor=true&cauthor_uid=24250812), [Chen FP](http://www.ncbi.nlm.nih.gov/pubmed?term=Chen FP%5BAuthor%5D&cauthor=true&cauthor_uid=24250812): **MiR-223 regulates human embryonic stem cell differentiation by targeting the IGF-1R/Akt signaling pathway**. [*PLoS One*](http://www.ncbi.nlm.nih.gov/pubmed/24250812) 2013,**8(11)**:e78769.

44. [Deng Y](http://www.ncbi.nlm.nih.gov/pubmed?term=Deng Y%5BAuthor%5D&cauthor=true&cauthor_uid=23768901), [Zhou H](http://www.ncbi.nlm.nih.gov/pubmed?term=Zhou H%5BAuthor%5D&cauthor=true&cauthor_uid=23768901), [Zou D](http://www.ncbi.nlm.nih.gov/pubmed?term=Zou D%5BAuthor%5D&cauthor=true&cauthor_uid=23768901), [Xie Q](http://www.ncbi.nlm.nih.gov/pubmed?term=Xie Q%5BAuthor%5D&cauthor=true&cauthor_uid=23768901), [Bi X](http://www.ncbi.nlm.nih.gov/pubmed?term=Bi X%5BAuthor%5D&cauthor=true&cauthor_uid=23768901), [Gu P](http://www.ncbi.nlm.nih.gov/pubmed?term=Gu P%5BAuthor%5D&cauthor=true&cauthor_uid=23768901), [Fan X](http://www.ncbi.nlm.nih.gov/pubmed?term=Fan X%5BAuthor%5D&cauthor=true&cauthor_uid=23768901): **The role of miR-31-modified adipose tissue-derived stem cells in repairing rat critical-sized calvarial defects**. [*Biomaterials*](http://www.ncbi.nlm.nih.gov/pubmed/23768901)

45. [Cui L](http://www.ncbi.nlm.nih.gov/pubmed?term=Cui L%5BAuthor%5D&cauthor=true&cauthor_uid=24232094), [Shi Y](http://www.ncbi.nlm.nih.gov/pubmed?term=Shi Y%5BAuthor%5D&cauthor=true&cauthor_uid=24232094), [Zhou X](http://www.ncbi.nlm.nih.gov/pubmed?term=Zhou X%5BAuthor%5D&cauthor=true&cauthor_uid=24232094), [Wang X](http://www.ncbi.nlm.nih.gov/pubmed?term=Wang X%5BAuthor%5D&cauthor=true&cauthor_uid=24232094), [Wang J](http://www.ncbi.nlm.nih.gov/pubmed?term=Wang J%5BAuthor%5D&cauthor=true&cauthor_uid=24232094), [Lan Y](http://www.ncbi.nlm.nih.gov/pubmed?term=Lan Y%5BAuthor%5D&cauthor=true&cauthor_uid=24232094), [Wang M](http://www.ncbi.nlm.nih.gov/pubmed?term=Wang M%5BAuthor%5D&cauthor=true&cauthor_uid=24232094), [Zheng L](http://www.ncbi.nlm.nih.gov/pubmed?term=Zheng L%5BAuthor%5D&cauthor=true&cauthor_uid=24232094), [Li H](http://www.ncbi.nlm.nih.gov/pubmed?term=Li H%5BAuthor%5D&cauthor=true&cauthor_uid=24232094), [Wu Q](http://www.ncbi.nlm.nih.gov/pubmed?term=Wu Q%5BAuthor%5D&cauthor=true&cauthor_uid=24232094), [Zhang J](http://www.ncbi.nlm.nih.gov/pubmed?term=Zhang J%5BAuthor%5D&cauthor=true&cauthor_uid=24232094), [Fan D](http://www.ncbi.nlm.nih.gov/pubmed?term=Fan D%5BAuthor%5D&cauthor=true&cauthor_uid=24232094), [Han Y](http://www.ncbi.nlm.nih.gov/pubmed?term=Han Y%5BAuthor%5D&cauthor=true&cauthor_uid=24232094): **A set of microRNAs mediate direct conversion of human umbilical cord lining-derived mesenchymal stem cells into hepatocytes**. [*Cell Death Dis*](http://www.ncbi.nlm.nih.gov/pubmed/24232094) 2013**,4**:e918.

46. [Giraud-Triboult K](http://www.ncbi.nlm.nih.gov/pubmed?term=Giraud-Triboult K%5BAuthor%5D&cauthor=true&cauthor_uid=21081659), [Rochon-Beaucourt C](http://www.ncbi.nlm.nih.gov/pubmed?term=Rochon-Beaucourt C%5BAuthor%5D&cauthor=true&cauthor_uid=21081659), [Nissan X](http://www.ncbi.nlm.nih.gov/pubmed?term=Nissan X%5BAuthor%5D&cauthor=true&cauthor_uid=21081659), [Champon B](http://www.ncbi.nlm.nih.gov/pubmed?term=Champon B%5BAuthor%5D&cauthor=true&cauthor_uid=21081659), [Aubert S](http://www.ncbi.nlm.nih.gov/pubmed?term=Aubert S%5BAuthor%5D&cauthor=true&cauthor_uid=21081659), [Piétu G](http://www.ncbi.nlm.nih.gov/pubmed?term=Piétu G%5BAuthor%5D&cauthor=true&cauthor_uid=21081659): **Combined mRNA and microRNA profiling reveals that miR-148a and miR-20b control human mesenchymal stem cell phenotype via EPAS1.** [*Physiol Genomics*](http://www.ncbi.nlm.nih.gov/pubmed/21081659)2011,**43(2)**:77-86.

47. [Yu J](http://www.ncbi.nlm.nih.gov/pubmed?term=Yu J%5BAuthor%5D&cauthor=true&cauthor_uid=23554686), [Li Q](http://www.ncbi.nlm.nih.gov/pubmed?term=Li Q%5BAuthor%5D&cauthor=true&cauthor_uid=23554686), [Xu Q](http://www.ncbi.nlm.nih.gov/pubmed?term=Xu Q%5BAuthor%5D&cauthor=true&cauthor_uid=23554686), [Liu L](http://www.ncbi.nlm.nih.gov/pubmed?term=Liu L%5BAuthor%5D&cauthor=true&cauthor_uid=23554686), [Jiang B](http://www.ncbi.nlm.nih.gov/pubmed?term=Jiang B%5BAuthor%5D&cauthor=true&cauthor_uid=23554686): **MiR-148a inhibits angiogenesis by targeting ERBB3.** *J Biomed Res* 2011,**25(3):**170-7.

48. [Qiao Y](http://www.ncbi.nlm.nih.gov/pubmed?term=Qiao Y%5BAuthor%5D&cauthor=true&cauthor_uid=21893058), [Ma N](http://www.ncbi.nlm.nih.gov/pubmed?term=Ma N%5BAuthor%5D&cauthor=true&cauthor_uid=21893058), [Wang X](http://www.ncbi.nlm.nih.gov/pubmed?term=Wang X%5BAuthor%5D&cauthor=true&cauthor_uid=21893058), [Hui Y](http://www.ncbi.nlm.nih.gov/pubmed?term=Hui Y%5BAuthor%5D&cauthor=true&cauthor_uid=21893058), [Li F](http://www.ncbi.nlm.nih.gov/pubmed?term=Li F%5BAuthor%5D&cauthor=true&cauthor_uid=21893058), [Xiang Y](http://www.ncbi.nlm.nih.gov/pubmed?term=Xiang Y%5BAuthor%5D&cauthor=true&cauthor_uid=21893058), [Zhou J](http://www.ncbi.nlm.nih.gov/pubmed?term=Zhou J%5BAuthor%5D&cauthor=true&cauthor_uid=21893058), [Zou C](http://www.ncbi.nlm.nih.gov/pubmed?term=Zou C%5BAuthor%5D&cauthor=true&cauthor_uid=21893058), [Jin J](http://www.ncbi.nlm.nih.gov/pubmed?term=Jin J%5BAuthor%5D&cauthor=true&cauthor_uid=21893058), [Lv G](http://www.ncbi.nlm.nih.gov/pubmed?term=Lv G%5BAuthor%5D&cauthor=true&cauthor_uid=21893058), [Jin H](http://www.ncbi.nlm.nih.gov/pubmed?term=Jin H%5BAuthor%5D&cauthor=true&cauthor_uid=21893058), [Gao X](http://www.ncbi.nlm.nih.gov/pubmed?term=Gao X%5BAuthor%5D&cauthor=true&cauthor_uid=21893058): **MiR-483-5p controls angiogenesis in vitro and targets serum response factor**. [*FEBS Lett*](http://www.ncbi.nlm.nih.gov/pubmed/21893058) 2011, **585(19)**:3095-100.

49. [Smigielska-Czepiel K](http://www.ncbi.nlm.nih.gov/pubmed?term=Smigielska-Czepiel K%5BAuthor%5D&cauthor=true&cauthor_uid=24401767), [van den Berg A](http://www.ncbi.nlm.nih.gov/pubmed?term=van den Berg A%5BAuthor%5D&cauthor=true&cauthor_uid=24401767), [Jellema P](http://www.ncbi.nlm.nih.gov/pubmed?term=Jellema P%5BAuthor%5D&cauthor=true&cauthor_uid=24401767), [van der Lei RJ](http://www.ncbi.nlm.nih.gov/pubmed?term=van der Lei RJ%5BAuthor%5D&cauthor=true&cauthor_uid=24401767), [Bijzet J](http://www.ncbi.nlm.nih.gov/pubmed?term=Bijzet J%5BAuthor%5D&cauthor=true&cauthor_uid=24401767), [Kluiver J](http://www.ncbi.nlm.nih.gov/pubmed?term=Kluiver J%5BAuthor%5D&cauthor=true&cauthor_uid=24401767), [Boots AM](http://www.ncbi.nlm.nih.gov/pubmed?term=Boots AM%5BAuthor%5D&cauthor=true&cauthor_uid=24401767), [Brouwer E](http://www.ncbi.nlm.nih.gov/pubmed?term=Brouwer E%5BAuthor%5D&cauthor=true&cauthor_uid=24401767), [Kroesen BJ](http://www.ncbi.nlm.nih.gov/pubmed?term=Kroesen BJ%5BAuthor%5D&cauthor=true&cauthor_uid=24401767):**Comprehensive analysis of miRNA expression in T-cell subsets of rheumatoid arthritis patients reveals defined signatures of naive and memory Tregs.** [*Author information Genes Immun*](http://www.ncbi.nlm.nih.gov/pubmed/24401767)2014,**15(2)**:115-25.

50. [Papagregoriou G](http://www.ncbi.nlm.nih.gov/pubmed?term=Papagregoriou G%5BAuthor%5D&cauthor=true&cauthor_uid=22319602), [Erguler K](http://www.ncbi.nlm.nih.gov/pubmed?term=Erguler K%5BAuthor%5D&cauthor=true&cauthor_uid=22319602), [Dweep H](http://www.ncbi.nlm.nih.gov/pubmed?term=Dweep H%5BAuthor%5D&cauthor=true&cauthor_uid=22319602), [Voskarides K](http://www.ncbi.nlm.nih.gov/pubmed?term=Voskarides K%5BAuthor%5D&cauthor=true&cauthor_uid=22319602), [Koupepidou P](http://www.ncbi.nlm.nih.gov/pubmed?term=Koupepidou P%5BAuthor%5D&cauthor=true&cauthor_uid=22319602), [Athanasiou Y](http://www.ncbi.nlm.nih.gov/pubmed?term=Athanasiou Y%5BAuthor%5D&cauthor=true&cauthor_uid=22319602), [Pierides A](http://www.ncbi.nlm.nih.gov/pubmed?term=Pierides A%5BAuthor%5D&cauthor=true&cauthor_uid=22319602), [Gretz N](http://www.ncbi.nlm.nih.gov/pubmed?term=Gretz N%5BAuthor%5D&cauthor=true&cauthor_uid=22319602), [Felekkis KN](http://www.ncbi.nlm.nih.gov/pubmed?term=Felekkis KN%5BAuthor%5D&cauthor=true&cauthor_uid=22319602), [Deltas C](http://www.ncbi.nlm.nih.gov/pubmed?term=Deltas C%5BAuthor%5D&cauthor=true&cauthor_uid=22319602): **A miR-1207-5p binding site polymorphism abolishes regulation of HBEGF and is associated with disease severity in CFHR5 nephropathy**. [*PLoS One*](http://www.ncbi.nlm.nih.gov/pubmed/22319602) 2012,**7(2)**:e31021.

51. [Hu R](http://www.ncbi.nlm.nih.gov/pubmed?term=Hu R%5BAuthor%5D&cauthor=true&cauthor_uid=21324897), [Liu W](http://www.ncbi.nlm.nih.gov/pubmed?term=Liu W%5BAuthor%5D&cauthor=true&cauthor_uid=21324897), [Li H](http://www.ncbi.nlm.nih.gov/pubmed?term=Li H%5BAuthor%5D&cauthor=true&cauthor_uid=21324897), [Yang L](http://www.ncbi.nlm.nih.gov/pubmed?term=Yang L%5BAuthor%5D&cauthor=true&cauthor_uid=21324897), [Chen C](http://www.ncbi.nlm.nih.gov/pubmed?term=Chen C%5BAuthor%5D&cauthor=true&cauthor_uid=21324897), [Xia ZY](http://www.ncbi.nlm.nih.gov/pubmed?term=Xia ZY%5BAuthor%5D&cauthor=true&cauthor_uid=21324897), [Guo LJ](http://www.ncbi.nlm.nih.gov/pubmed?term=Guo LJ%5BAuthor%5D&cauthor=true&cauthor_uid=21324897), [Xie H](http://www.ncbi.nlm.nih.gov/pubmed?term=Xie H%5BAuthor%5D&cauthor=true&cauthor_uid=21324897), [Zhou HD](http://www.ncbi.nlm.nih.gov/pubmed?term=Zhou HD%5BAuthor%5D&cauthor=true&cauthor_uid=21324897), [Wu XP](http://www.ncbi.nlm.nih.gov/pubmed?term=Wu XP%5BAuthor%5D&cauthor=true&cauthor_uid=21324897), [Luo XH](http://www.ncbi.nlm.nih.gov/pubmed?term=Luo XH%5BAuthor%5D&cauthor=true&cauthor_uid=21324897): **A Runx2/miR-3960/miR-2861 regulatory feedback loop during mouse osteoblast differentiation.** [*J Biol Chem*](http://www.ncbi.nlm.nih.gov/pubmed/21324897) 2011,**286(14)**:12328-39.

52. [Zhang X](http://www.ncbi.nlm.nih.gov/pubmed?term=Zhang X%5BAuthor%5D&cauthor=true&cauthor_uid=22997154), [Chen C](http://www.ncbi.nlm.nih.gov/pubmed?term=Chen C%5BAuthor%5D&cauthor=true&cauthor_uid=22997154), [Wu M](http://www.ncbi.nlm.nih.gov/pubmed?term=Wu M%5BAuthor%5D&cauthor=true&cauthor_uid=22997154), [Chen L](http://www.ncbi.nlm.nih.gov/pubmed?term=Chen L%5BAuthor%5D&cauthor=true&cauthor_uid=22997154), [Zhang J](http://www.ncbi.nlm.nih.gov/pubmed?term=Zhang J%5BAuthor%5D&cauthor=true&cauthor_uid=22997154), [Zhang X](http://www.ncbi.nlm.nih.gov/pubmed?term=Zhang X%5BAuthor%5D&cauthor=true&cauthor_uid=22997154), [Zhang Z](http://www.ncbi.nlm.nih.gov/pubmed?term=Zhang Z%5BAuthor%5D&cauthor=true&cauthor_uid=22997154), [Wu J](http://www.ncbi.nlm.nih.gov/pubmed?term=Wu J%5BAuthor%5D&cauthor=true&cauthor_uid=22997154), [Wang J](http://www.ncbi.nlm.nih.gov/pubmed?term=Wang J%5BAuthor%5D&cauthor=true&cauthor_uid=22997154), [Chen X](http://www.ncbi.nlm.nih.gov/pubmed?term=Chen X%5BAuthor%5D&cauthor=true&cauthor_uid=22997154), [Huang T](http://www.ncbi.nlm.nih.gov/pubmed?term=Huang T%5BAuthor%5D&cauthor=true&cauthor_uid=22997154), [Chen L](http://www.ncbi.nlm.nih.gov/pubmed?term=Chen L%5BAuthor%5D&cauthor=true&cauthor_uid=22997154), [Yuan Z](http://www.ncbi.nlm.nih.gov/pubmed?term=Yuan Z%5BAuthor%5D&cauthor=true&cauthor_uid=22997154): **Plasma microRNA profile as a predictor of early virological response to interferon treatment in chronic hepatitis B patients.** *Antivir Ther* 2012,**17(7)**:1243-53.

53. [Lu J](http://www.ncbi.nlm.nih.gov/pubmed?term=Lu J%5BAuthor%5D&cauthor=true&cauthor_uid=22295894), [Kwan BC](http://www.ncbi.nlm.nih.gov/pubmed?term=Kwan BC%5BAuthor%5D&cauthor=true&cauthor_uid=22295894), [Lai FM](http://www.ncbi.nlm.nih.gov/pubmed?term=Lai FM%5BAuthor%5D&cauthor=true&cauthor_uid=22295894), [Tam LS](http://www.ncbi.nlm.nih.gov/pubmed?term=Tam LS%5BAuthor%5D&cauthor=true&cauthor_uid=22295894), [Li EK](http://www.ncbi.nlm.nih.gov/pubmed?term=Li EK%5BAuthor%5D&cauthor=true&cauthor_uid=22295894), [Chow KM](http://www.ncbi.nlm.nih.gov/pubmed?term=Chow KM%5BAuthor%5D&cauthor=true&cauthor_uid=22295894), [Wang G](http://www.ncbi.nlm.nih.gov/pubmed?term=Wang G%5BAuthor%5D&cauthor=true&cauthor_uid=22295894), [Li PK](http://www.ncbi.nlm.nih.gov/pubmed?term=Li PK%5BAuthor%5D&cauthor=true&cauthor_uid=22295894), [Szeto CC](http://www.ncbi.nlm.nih.gov/pubmed?term=Szeto CC%5BAuthor%5D&cauthor=true&cauthor_uid=22295894): **Glomerular and tubulointerstitial miR-638, miR-198 and miR-146a expression in lupus nephritis.** [*Nephrology (Carlton)*](http://www.ncbi.nlm.nih.gov/pubmed/22295894) 2012,**17(4)**:346-51.
